# Development of Antibacterial and Antifungal Triazole Chromium(III) and Cobalt(II) Complexes: Synthesis and Biological Activity Evaluations

**DOI:** 10.3390/molecules23082013

**Published:** 2018-08-13

**Authors:** Ricardo A. Murcia, Sandra M. Leal, Martha V. Roa, Edgar Nagles, Alvaro Muñoz-Castro, John J. Hurtado

**Affiliations:** 1 Departament of Chemistry, Universidad de los Andes, Carrera 1 No. 18A-12, 111711 Bogotá, Colombia; ra.murcia@uniandes.edu.co; 2 Programa de Bacteriología y Laboratorio Clínico, Facultad de Ciencias de la Salud, Universidad de Santander, 680002 Bucaramanga, Colombia; sa.leal@mail.udes.edu.co (S.M.L.); ma.roa@mail.udes.edu.co(M.V.R.); 3 Programa de Maestría en Investigación en Enfermedades Infecciosas, Facultad de Ciencias de la Salud, Universidad de Santander, 680002 Bucaramanga, Colombia; 4 Facultad de Ciencias Naturales y Matemáticas, Universidad de Ibagué, Carrera 22 Calle 67, 730001 Ibagué, Colombia; edgar.nagles@unibague.edu.co; 5 Grupo de Química Inorgánica y Materiales Moleculares, Universidad Autonoma de Chile, El Llano Subercaseaux, 2801 Santiago, Chile; alvaro.munoz@uautonoma.cl

**Keywords:** triazole ligands, cobalt(II) and chromium(III) complexes, antibacterial activity, antifungal activity

## Abstract

In this work, six complexes (**2**–**7**) of Cr(III) and Co(II) transition metals with triazole ligands were synthesized and characterized. In addition, a new ligand, 3,5-bis(1,2,4-triazol-1-ylmethyl)toluene (**1**), was synthesized and full characterized. The complexes were obtained as air-stable solids and characterized by melting point, electrical conductivity, thermogravimetric analysis, and Raman, infrared and ultraviolet/visible spectroscopy. The analyses and spectral data showed that complexes **3**–**7** had 1:1 (*M*:*L*) stoichiometries and octahedral geometries, while **2** had a 1:2 (*M*:*L*) ratio, which was supported by DFT calculations. The complexes and their respective ligands were evaluated against bacterial and fungal strains with clinical relevance. All the complexes showed higher antibacterial and antifungal activities than the free ligands. The complexes were more active against fungi than against bacteria. The activities of the chromium complexes against *Candida tropicalis* are of great interest, as they showed minimum inhibitory concentration 50 (MIC_50_) values between 7.8 and 15.6 μg mL^−1^. Complexes **5** and **6** showed little effect on Vero cells, indicating that they are not cytotoxic. These results can provide an important platform for the design of new compounds with antibacterial and antifungal activities.

## 1. Introduction

Bacteria and fungi, due to their high rate of development and adaptation, develop resistance to currently used drugs for the treatment of infectious diseases, leading to global public health problems [1,2,3,4,5,6,7,8,9,10,11]. Azole-derived compounds have been widely used for the treatment of fungal diseases due to their properties including broad spectrum of action, chemical stability and oral bioavailability. The mechanism of action of azoles against fungi is based on the inhibition of ergosterol [12]. Compared to azole derivatives, triazole compounds exhibit higher antifungal activity and wider spectrum of action [13]. Azoles also show antibacterial activity, inhibiting the enoyl acyl carrier protein reductase [14]. Furthermore, triazole complexes showed very good antimicrobial [15] and antitumor [16] activities. The combination of metals and azoles is a promising strategy to develop new efficient drugs, even against drug-resistant pathogens [17,18,19,20,21,22]. The development of these new drugs is related to the complexation of azole derivatives with transition metals that have low toxicity and are biocompatible [18,23,24]. Previous studies demonstrated that the resulting complexes may have higher antimicrobial activity than the corresponding free azole ligands, and, in some cases, exceeding that of standard test substances (nystatin) [25,26]. In this context, we previously studied the antibacterial and antifungal activities of cobalt(II) and copper(II) complexes derived from benzotriazole, which showed higher antimicrobial activities than the free ligands [24,27]. In line with our results, Sumrra et al. recently reported that complexes derived from triazole had antibacterial and antifungal activities higher than the corresponding ligands [28]. Two well-known theories have been presented to explain the increase in the activity in the complexes with respect to the free ligands (the overtone’s concept [17] and Tweedy’s chelation theory [29]). The coordination of triazoles to metal centers could be an interesting approach for achieving antibacterial and antifungal activities with lower concentrations of the complexes. In this work, we report the synthesis and characterization of new chromium(III) and cobalt(II) complexes derived from triazole ligands and their antibacterial, antifungal and cytotoxic activities.

## 2. Discussion

### 2.1. Synthesis and Characterization of Ligand *(**1**)*

**Scheme 1:**
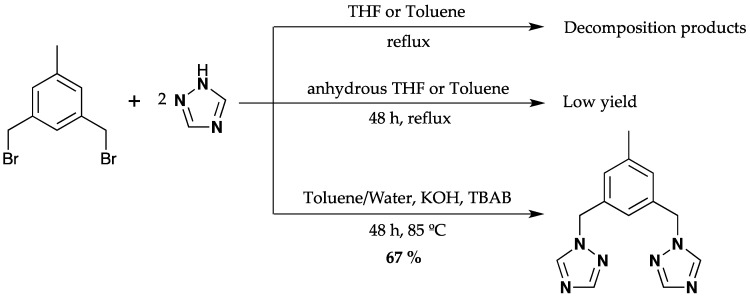
Synthesis of the ligand (**1**).

Initially, we tried to prepare the ligand 3,5-bis(1,2,4-triazol-1-ylmethyl)toluene by the direct reaction of 1,3-bis(bromomethyl)toluene with 1*H*-1,2,4-triazole and using THF and toluene (not anhydrous) as solvents, we obtained a mixture of decomposition products observed by NMR, which were not characterized. Later, the reaction was carried out in an inert atmosphere (nitrogen) using standard Schlenk techniques and anhydrous toluene, which afforded **1** in low yield (5%). Then, the ligand was prepared by a phase-transfer catalyzed reaction of 1,3-bis(bromomethyl)toluene with 1*H*-1,2,4-triazole followed by thin-layer chromatography (Scheme 1).

The ligand was isolated as a white, air-stable solid in high yield. It was characterized by elemental analysis, mass spectrometry, and ^1^H and ^13^C-NMR, FTIR, UV-Visible and Raman spectroscopy. The ^1^H and ^13^C-NMR chemical shifts were assigned with the aid of DEPT 135 and HSQC experiments. The ^1^H-NMR spectrum show the methylene groups (CH_2_) between the central aromatic ring and the triazoles at 5.35 ppm, and the methyl group (CH_3_) of the toluene appears at 2.25 ppm. The three protons of toluene ring were observed between 7.02 and 8.63 ppm. This assignment was confirmed by the integrals of the peaks (Figures S3–S6 in the Supplementary Materials). The solid-state FTIR spectrum showed bands between 1346 and 1608 cm^−1^. The bands at 1504 and 1384 cm^−1^ were assigned to the stretching of the C=C and C=N bonds, respectively [30] (Figure S14 in the Supplementary Materials). 

### 2.2. Synthesis and Characterization of Metal Complexes

**Figure 1:**
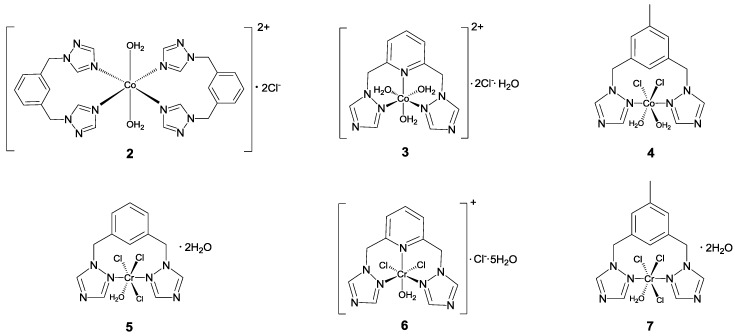
Possible structures of the complexes under study.

The key to the synthesis of these complexes was the choice of a suitable solvent in each reaction (Figure 1). The reactions were carried out in acetone, which dissolved both the ligand and the starting saltsbut did not solubilize the final complex. In all cases, almost immediate precipitation of the complexes was observed, and the products were washed with acetone. 

In the case of chromium complexes **5**–**7**, reactions similar to those used to obtain the cobalt complexes were carried out. When the respective ligand was mixed with the chromium salt, **5**–**7** immediately precipitated. These complexes were isolated as green, non-hygroscopic, air-stable solids.

Electrical conductivity measurements were made for all the complexes and starting salts of each metal using DMSO as the solvent. For the cobalt salt, a conductivity of 48.0 µS was obtained, and the conductivity of the chromium salt was 40.2 µS. When comparing these values with those obtained for the cobalt complexes, the conductivities of **2** (52.9 µS) and **3** (50.5 µS) were higher (Table 1). 

This result is probably due to the electron mobility in the π-conjugated systems of the ligands and to the presence of ionic species.

On the other hand, **4** showed a lower conductivity than that of the free cobalt salt, which indicates that this complex does not exist as electrolytes in solution [31]. The conductivity values observed for **5**–**7** were lower than that obtained with the initial chromium salt, which may be associated with the absence of ionic species. Although, it has been reported that showing conductivity values between 23 and 42 µS when dissolved in DMSO is characteristic of electrolyte complexes with 1:1 ratios [31]. Taking into account the conductivity results, complexes **2**, **3** and **6** are probably electrolytes containing chlorides as the counterions.

#### 2.2.1. FTIR Spectroscopy 

**Figure 2:**
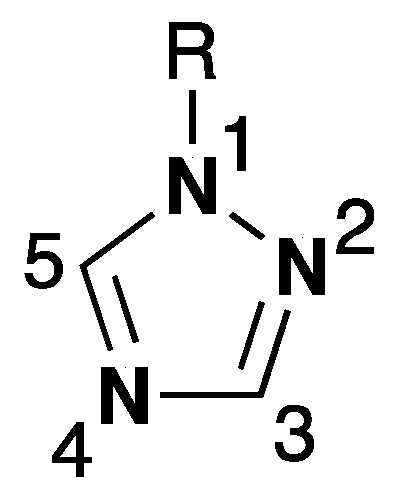
Atom numbering for signal assignment for 1,2,4-triazole.

The obtained complexes were analyzed by infrared spectroscopy to observe the shifts of certain bands relative to those of the free ligands following coordination to the metal center. (Table S1 in the Supplementary Materials) summarizes the assignment of the important bands in the complexes compared to those of the free ligands. Figure 2 shows the structure of 1*H*-1,2,4-triazolewith its respective atom numbering to facilitate the discussion of the spectra.

In the case of **L1** and its complexes (**2** and **5**), a shift in the band associated with the stretching of the bond (N-C) between a carbon and a nitrogen atom of the free 1,2,4-triazole was observed [32]; for **L1**, the band was at 1138 cm^−1^, while for **2** and **5** this band is shift to lower wavenumbers (1128 cm^−1^ and 1123 cm^−1^, respectively).

Such results indicate greater rigidity in the chemical bonds in the all complexes confirming the coordination of the metals to the N of the azole [27,33]. Bands are also observed at 3435 and 3429 cm^−1^ for **2** and **5**, respectively (Figures S9 and S10 in Supplementary Materials), that correspond to the stretches of the bond (O-H) in water molecules [34]. Due to the intensity and width of these bands, it can be inferred that in **2**, the H_2_O molecules are coordinated to the metal center, while for the chromium complex (**5**), the band is associated with waters of hydration.

For **L2** and its complexes (**3** and **6**), were observed three interesting bands that can give information on how the ligand is coordinated to the metal center. First, a shift of the C-N band of the triazole was observed [32]; in the free ligand it appears at 1141 cm^−1^, while for **3** and **6** it appears at 1125 cm^−1^. Additionally, a second band that appears in the spectrum of the free ligand at 1273 cm^−1^ and was assigned to the N-N bond of the triazole was observed in the spectra of **3** and **6** at 1277 and 1283 cm^−1^, respectively (Figures S12 and S13 in the Supplementary Materials). This result confirms the possible coordination to the metal center via N2 of the triazole. The third band of interest is associated with the stretching N=C of the pyridine [35]; it was observed in the spectrum of **L2** at 1512 cm^−1^ and at 1524 and 1533 cm^−1^ in those of **3** and **6**, respectively. This allows us to evaluate the coordination of the nitrogen of the pyridine to the metal center. Bands associated with waters molecules of hydration were also found at 3402 cm^−1^ (**3**) and 3379 cm^−1^ (**6**). (Calculated at 3430 and 3354 cm^−1^, respectively, from DFT calculations). A slight shift in the band associated with the tension of the triazole ring in **1** (Exp. 891 cm^−1^; Calc. 830 cm^−1^) was observed compared with the same bands of complexes **4** and **7** (Exp. 881 and 887 cm^−1^, respectively, Figures S15 and S16 in the Supplementary Materials; Calc. 840 and 833 cm^−1^), indicating a decrease in the rigidity of this ring. The band indicative of the C-N bond of the triazole in **1** is observed at 1134 cm^−1^, and for **4** and **7** the band is shifted to lower wavenumbers (1128 and 1125 cm^−1^, respectively, Calc. 1089 and 1065 cm^−1^) due to the decrease in the vibrational energy of this bond by the coordination of N2 of the triazole to the metal center. It was also possible to observe a shift to greater wavenumber in the N-N triazole band, which for the free ligand (**1**) appears at 1261 cm^−1^ and for the complexes appears at 1279 cm^−1^ (**4**) and 1285 cm^−1^ (**7**) (Calculated at 1240 and 1232 cm^−1^). In the spectra of these complexes, bands corresponding to the bond (O-H) in water molecules are observed at 3412 cm^−1^ (**4**) and 3375 cm^−1^ (**7**) [34], and due to the intensity and width of these bands, it can be inferred that in **4**, the H_2_O molecules are coordinated to the metal center, while for **9**, the band is associated with watersmolecules of hydration. 

It should be noted that for all complexes (**2**–**7**), a band associated with the M-OH_2_ bond was observed between 1600–1630 cm^−1^ [35], and this result gives an indication of the presence of coordination water molecules.

#### 2.2.2. Raman Spectroscopy 

Triazole, ligands and the obtained complexes were analyzed by Raman spectroscopy to observe the shifts in the bands of free ligands following coordination to the metal center (Supplementary Materials).

In the complexes, some bands assigned to the N-H bond were not observed at 1481, 977 and 918 cm^−1^, assigned to the N-H bond indicating **L1**, **2** and **5** were successfully prepared.

Bands were observed in the zone between 1500 and 900 cm^−1^ in the spectra of **L1**, **2** and **5**, and these bands corresponded to the N-C bond of the triazole [36,37]. These bands are shifted towards lower wavenumbers in the spectra of the complexes, which indicated a decrease in the rigidity of the triazolic ring and confirmed the coordination of the metal to the N of the triazole. In addition, the bands at 1431 and 1171 cm^−1^ in the spectrum of **L1** did not show considerable shifting in the spectra of the complexes.

On the other hand, the bands observed at 274 cm^−1^ (**3**) and 266 cm^−1^ (**6**) are assigned to the vibration (O-H) of both coordinated water and watersmolecules of hydration.

For **L2** and its complexes **3** and **6**, was observed an interesting band from the N-N bond, which can indicate how the ligand is coordinated to the metal center [36]. This band appears to shift to higher wavenumbers in the spectra of the complexes compared to the that of the free ligand. This result suggests the coordination of the metal to N2 of the triazole. In addition, the band at 1423 cm^−1^ associated with the stretching of the C=N bond of the pyridine in **L2** is shifted to 1430 cm^−1^ (**3**) and 1425 cm^−1^ (**6**), indicating the coordination of the nitrogen of the pyridine to the metal center.

In the spectra of ligand **1** and its complexes (**4** and **7**), we observed shifts in some of the bands. These bands appear in the spectrum of **1** at 1496 cm^−1^ and 533 cm^−1^, and in that of **4**, they are at 1464 cm^−1^ and 522 cm^−1^, and in that of **7**, they are at 1472 cm^−1^ and 518 cm^−1^, respectively. These bands are due to the N-C bond of the triazole and confirmed the coordination of the metal to the triazole

Two bands of interest were observed at 1370 and 1013 cm^−1^ for **1**, and they shift to 1358 and 996 cm^−1^ in the spectrum of **4**, and at 1355 cm^−1^ and 997 cm^−1^ in that of **7**. The first band is assigned to the N-C bond and the second to the N-N bond of the triazole, and these bands indicate that the coordination is via N2 of the triazole.

#### 2.2.3. UV/Vis Spectroscopy

The UV-Vis spectra were recorded in DMSO to study the electronic properties of the complexes and to relate the properties to the possible structures. Table 2 shows some of the bands for the starting salts, ligands and complexes **2**–**7** that were observed in their UV/Vis spectra. For all complexes, one or two bands between 200 and 300 nm corresponding to the transitions between the π-π* orbitals of the ligand were observed. In addition, for the complexes, the most intense bands were observed in the UV zone and were not shifted relative to the bands of the free ligand, and they only increased in intensity due to the interaction of the orbitals of the ligand with those of the metal.

In the spectra of the cobalt(II) salt and its complexes (**2**–**4**), bands at 600–700 nm were observed, and these bands may be assigned to the *^4^T*_1*g*_ (F) ← *^4^T*_1*g*_ (P) transitions in a distorted octahedral geometry in solution [38].

Chromium complexes **5**–**7** had a green color in both the solid and solution phases. These compounds showed intense bands in the UV region of their spectra, whereas in the region between 400 and 500 nm, no bands were observed. In comparing the spectra of cobalt(II) complex **6** and **L2**, a small hypsochromic shift was observed. This can be explained by the charge transfer transitions of the metal to the ligand [27,33].

#### 2.2.4. Thermal Analysis

The stages of decomposition and temperature ranges of the complexes are given in (Table S5 in the Supplementary Materials). All results are proposed based the probable mass losses because detection of the decomposition products was not possible. For all the complexes, a final residue containing metal and chlorine was observed. In addition, the thermograms show a decomposition temperature above 600 °C. TGA and derivative thermogravimetric analysis data are shown in the Supplementary Materials. In the case of **2**–**4**, the loss of an organic fragment was found at temperatures greater than 200 °C.

For **2**, a weight reduction of 44% was observed at 238–342 °C, which implies a loss of **L1** plus two equivalents of H_2_O (42.74% calculated). It is proposed that these water molecules were bound to the metal center due to the high temperature required to remove them and based on the bands observed in the IR spectrum (3300–3600 cm^−1^). Subsequently, a second loss at 518 °C is attributed to the loss of a second fraction of the ligand, and finally, a third loss occurs leaving a metallic residue. In the case of complex **3**, the loss of a water molecule not coordinated to the metal center was observed to 58 °C, which is consistent with the IR spectrum. A second loss of 27% was observed and assigned to one ligand molecule (**L2**), and the remaining material existed as a residue of CoCNCl_2_. The TGA of **4,** initially showed a loss between 270–352 °C, which may correspond to the loss of two water molecules coordinated to the metal center because high temperatures were required for these losses to occur. Similar to the copper complexes, in the analysis of chromium complexes **5**–**7**, it was found that a temperature greater than 200 °C was necessary to lose an organic fragment. For all these complexes, the presence of both coordinating waters and watersmolecules of hydration was observed. An initial loss of H_2_O at 68 °C was observed for complex **5**, and this loss correspond to watersmolecules of hydration. Then, the loss of an organic fragment at 330 °C was observed, and the remaining material formed a residue of CrCl_3_. For **6**, only two mass losses are observed; an initial loss of 4H_2_O (watersmolecules of hydration) at 63 °C and a second loss of 71% was observed and assigned to the ligand molecule (**L2**), with the remained existing as a metallic residue. In complex **7**, three main losses were observed. The first one was assigned to the elimination of 3H_2_O, which occurs in two steps based on the two maximum DTG peaks that occur; it is proposed that, of these three molecules, two are watersmolecules of hydration and the third is coordinated. Subsequently, the elimination of most of the organic matter occurs. Finally, the remaining organic fraction (which is proposed to be the methyl of the toluene fragment present in the ligand) is lost generating CrCl_3_ residue.

### 2.3. Biological Activity

The biological activities of the ligands and metal complexes were evaluated against all the strains in three replicates. Combinations of ligands and metal salts did not exhibit antimicrobial activity. Table 3 shows the antibacterial, antifungal and cytotoxic activities of the ligands and complexes as determined by the broth microdilution method (MIC; μgmL^−1^) and colorimetric method (CC_50_; μgmL^−1^).

#### 2.3.1. Antibacterial Activity

The antibacterial activities of the ligands and their complexes were studied against *Staphylococcus aureus*, *Salmonella typhimurium and Escherichia coli*. The ligands, metal complexes and solvent control (DMSO) were screened separately for their antibacterial activities at concentrations of 2000–1.95 μg mL^−1^. Standards drugs (ampicillin, gentamicin, amphotericin B and itraconazole) were evaluated following the standars recommended by the CLSI. Bacterial species were more resistant to treatments with the new complexes. However, cobalt complexes **3** and **4** showed higher antibacterial activities with MIC values of 250 and 500 μg mL^−1^, respectively, for *S. aureus* (Table 3). In addition, these complexes have higher activities than free ligands **L1**, **L2** and **1**. This finding is likely related to the better lipophilicity of the complexes, cells and slowed the normal cellular processes of the microorganisms, resulting in increased antimicrobial activities or chelating effects [23,24,39,40]. We studied the antibacterial activities of twelve complexes derived from azole that contained cobalt and copper. In previous studies carried out in our research group, it was found that metal complexes containing copper and cobalt displayed better antibacterial effects against bacterial strains than those containing zinc [27].

Some studies have shown that azoles in complexes can inhibit the bacterial DNA [41]. For **3** and **4**, similar activities were observed, and this result is probably due to the central ring separator (benzene (**2**), pyridine (**3**) and toluene (**4**)) as the presence of toluene increased the fluidity of Gram-negative cell membranes [27]. The pyridine in the ligand increases the noncovalent interactions with DNA through intermolecular associations as it can form hydrogen bonds, which in turn increases the antibacterial activity [18,42,43]. Previous studies have shown that complexes of Co(II) and Cr(III) containing ligands derived from azoles present activities against *S. aureus* with MIC values between 80 and 500 μg mL^−1^ for Co(II) complexes [27,29,44,45] and 17 μg mL^−1^ for Cr(III) complexes [37]. Although only zone of inhibition results were shown [20,21,22].

#### 2.3.2. Antifungal Activity

The antifungal activities of the ligands and their complexes were studied against *Candida tropicalis*, *C. albicans* and *C. parasilopsis*. The compounds, amphotericin B, itraconazole and DMSO as the solvent control were screened separately for their antifungal activities at concentrations similar to those used in the antibacterial study. The free ligands showed higher value of MIC values than the complexes. The results of the antifungal activity tests suggested that all the synthesized metal complexes were effective in at least one of the strains used here, and they showed moderate MIC values compared to the reference drug amphotericin B. The cobalt complex showed MIC values between 31.25 and 250 μg mL^−1^, and those of the chromium complexes were 7.81–15.62 μg mL^−1^. This may be because azoles have higher antifungal activities [46,47]. Cobalt complexes have been studied as antimicrobial agents and have shown potential activities against different strains of fungi [27,48,49]. Nevertheless, there are few reports of studies against *C. tropicalis* using Co(II) complexes. Additionally, we concluded that these complexes do not show considerable activities as they show MIC values greater than 100 μg mL^−1^ [45,50,51,52,53]. The complexes of Co(II) (**2**–**4**) synthesized in this work showed better results. Moreover, **5**–**7** have higher antifungal activities than **2**–**4**, and the lack of reports on chromium(III) complexes with antifungal activity against *C. tropicalis* is notable. Further studies are necessary to elucidate the antifungal mechanism of these chromium complexes. In addition, we tested itraconazole as a reference drug against all the *Candida* species included in this study. Itraconazole showed higher antifungal activity than the complexes against *C. parasilopsis*, which was resistant to antimycotic control.

On the other hand, mammalian cells exposed to **5** and **6** did not show signs of cytotoxic effects. The complexes had little effect on Vero cells with CC_50_ values above of 300 μg mL^−1^, indicating that they presented low toxicity. Notably, although amphotericin B is more active than the complexes, it is a polyene that exhibits broad-spectrum antimicrobial effects, and it is highly nephrotoxic and thus its use is limited in immunocompromised patients [54]. In addition, the use of itraconazole is restricted specifically due to the emergence of resistant strains of *Candida*. 

### 2.4. Quantum-Chemical Calculations

**Figure 3:**
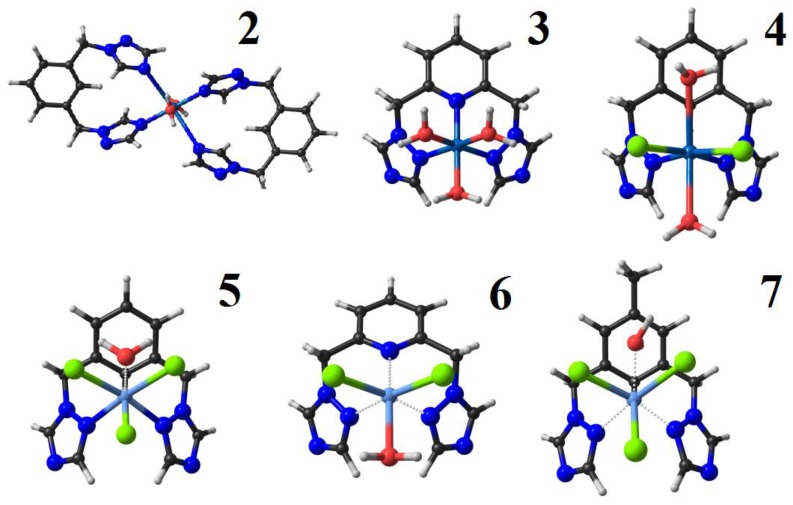
Optimized geometries for complexes **2**–**7**.

The relaxed geometries of the studied complexes (Figure 3) were obtained by density functional theory (DFT) calculations by using the ADF code [55] at the dispersion corrected BP86-D3 level of theory with all-electron TZ2P basis sets [56]. 

**Figure 4:**
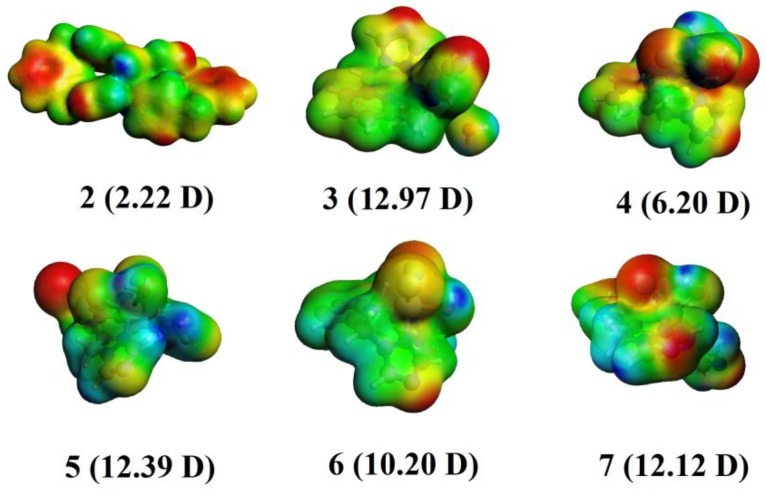
Molecular electrostatic potential maps of the studied complexes denoting their permanent dipole moments in Debye (D). Blue regions indicate charge depletion, and red regions indicate charge accumulation.

The evaluation of the relevant physicochemical properties to explain the observed differences in biological activity along the series exposed a key variation in the dipole moment, which has been early discussed as a useful parameter in drug-receptor interaction in the quantitative structure-activity relationship (QSAR) framework [57]. The permanent molecular dipole moment (µ) is a key factor in long-range electrostatic forces, which are relevant for structure stabilization and drug-site interactions in biomolecules [58]. In this sense, the calculated molecular dipole moment at the DFT level shows values ranging from 2.22 to 22.97 Debye (D), and these values represent the charge distribution over the whole structure as observed from the molecular electrostatic potential energy surface (MEP) (Figure 4). Interestingly, the lowest MIC values corresponded to larger permanent dipole moments (µ values), and in the studies on *C. tropicalis*, complexes **2** and **3** showed MIC values of 125 µg mL^−1^ and 31.25 µg mL^−^^1^, respectively, which are in line with the difference in their dipole moments (2.22 to 12.97 D, Figure 2) from an almost centrosymmetric (**2**) to a noncentrosymmetric system (**3**), respectively. For **4**, the MIC value in the same study was 62.50 µg mL^−1^, which appears to be connected to the decrease of in its dipole moment to 6.20 D relative to that of **3**. The same trend can be deduced from other biological traits, indicating that the dipole moment can be a useful parameter to consider during the further development of biologically active complexes.

## 3. Materials and Methods 

### 3.1. General Information

The starting salts CoCl_2_·6H_2_O and CrCl_3_·6H_2_O were used as received from Alfa Aesar (Ward Hill, MA, USA). The compounds 1*H*-1,2,4-triazole, 3,5-bis(bromomethyl)toluene, 1,3-bis(bromomethyl)benzene, tetrabutylammonium bromide (TBAB) were purchased from Sigma-Aldrich (St. Louis, MI, USA) and were used as received. 

1,3-Bis(1,2,4-triazol-1-ylmethyl)benzene (**L1**) [59] and 2,6-bis(1,2,4-triazol-1-ylmethyl)pyridine (**L2**) [60] were synthesized as described in the literature.

Elemental analysis (C, H and N) was performed with a Thermo Scientific™ FLASH 2000 CHNS/O Analyzer (Thermo Fisher Scientific, Waltham, MA, USA). Fourier transform infrared (FTIR) spectra were recorded on a Thermo Nicolet NEXUS FTIR spectrophotometer using KBr pellets. Melting points were determined on a Mel-Temp^®^ 1101D apparatus Electrothermal, Staffordshire, UK) in open capillary tubes and are uncorrected. Ultraviolet/visible (UV/vis) spectra were recorded on an Agilent Technologies Cary 100 spectrophotometer (Agilent Technologies, Santa Clara, CA, USA) in DMSO from 200 to 800 nm in a quartz cuvette with a path length of 1 cm. Molar conductivity measurements of the complexes were performed in a CON 700OAKTON instrument OAKTON Instruments, Vernon Hills, IL, USA) at 295 K (1 mM; dimethyl sulfoxide (DMSO)). Raman spectroscopy was performed in a HORIBA Yovin-Ivon spectrometer (HORIBA Scientific, Kyoto, Japan) using a laser with a wavelength of 786 nm. Thermogravimetric (TG) analyses of the complexes were conducted on a NETZSCH STA 409 PC/PG (NETZSCH, Selb, Bavaria, Germany) by evaluating 8–10 mg samples of the complexes in a nitrogen atmosphere. Samples were subjected to dynamic heating over a temperature range of 30–700 °C at a heating rate of 10 °C min^−1^. TG curves were analyzed to obtain the percent mass losses as a function of temperature. Nuclear magnetic resonance (NMR) spectra were recorded on a Bruker AscendTM-400 spectrometer (Bruker, Billerica, MA, USA) at 295 K. Chemical shifts are reported in ppm relative to SiMe_4_ (^1^H) as an internal standard. ^1^H and ^13^C-NMR chemical shifts (δ) are reported in parts per million (ppm) relative to TMS, with the residual solvent peak used as an internal reference; CDCl_3_ (^1^H-NMRδ: 7.26 and ^13^C-NMRδ: 77.2) and DMSO-*d*_6_ (^1^H-NMRδ: 2.50 and ^13^C-NMRδ: 39.5). High-resolution mass spectrometry (HRMS) data of ligand **1** was obtained on an Agilent Technologies Q-TOF 6520 spectrometer (Agilent Technologies, Santa Clara, CA, USA) *via* electrospray ionization (ESI) in positive ion modein chloroform.

### 3.2. Synthesis of 3,5-Bis(1,2,4-triazol-1-ylmethyl)toluene *(**1**)*

In a Schlenk tube equipped with a reflux condenser, 1*H*-1,2,4-triazole (1117 mg, 16.18 mmol), potassium hydroxide (1286 mg; 22.92 mmol), tetrabutylammonium bromide (405.7 mg; 1.26 mmol), and water (8 mL) were stirred at room temperature (r.t.) for 20 min. Then, 1,3-bis(bromomethyl)toluene (2279 mg; 8.20 mmol) and toluene (50 mL) were added, and the mixture was heated for 48 h at 85 °C. The resulting mixture was treated with water and the organic layer was separated and dried with magnesium sulfate. The solution was concentrated to dryness to give a yellow oil, which was purified by silica gel column chromatography (type 60) eluting with DCM:methanol 9:1 to give a pure compound (white solid). Yield 1167 g (67%). M.p.: 135–136 °C. FTIR (KBr, cm^−1^): 3090, 2924, 1609, 1504, 1466, 1431, 1385, 1346, 1261, 1207, 1134, 1018, 980 953, 918, 891, 745, 725, 567, 366, 351. Raman (cm^−1^): 1604, 1496, 1434, 1370, 1286, 1128, 1013, 760, 533, 282. 

**Figure 5:**
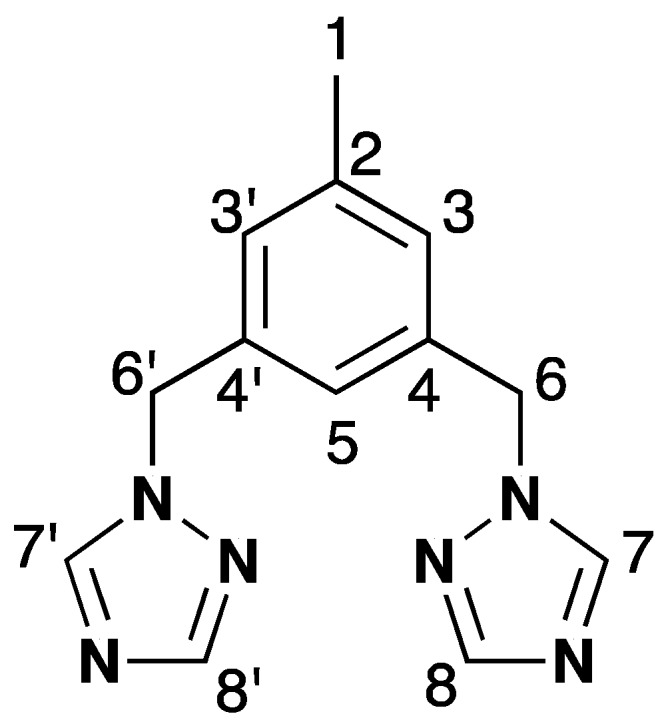
Atom numbering for signal assignment of the NMR for **1.**

Atom numbering for ligand **1** is as follows (Figure 5):

^1^H-NMR (400.1 MHz, DMSO-*d*_6_) δ 8.63 (s, 2H, 7, 7’), 7,97 (s, 2H, 8, 8’), 7,02 (s, 2H, 3, 3’), 7,00 (s, 1H, 5), 5,35 (s, 4H, 6, 6’), 2,25 (s, 3H, 1). ^13^C-NMR (DMSO-*d_6_*, 101 MHz) δ 151,65 (C8, C8’), 144,16 (C7, C7’), 138,31 (C2), 136,62 (C4, C4’), 127,97 (C3, C3’), 124,38 (C5), 51,81 (C6, C6’), 20,77 (C1). 

MS-ESI (*m*/*z*, ES^+^) calcd. For [M + H]^+^: 253; found: 253. UV/Vis (bands λ_max_, nm (ε, L mol^−1^ cm^−1^): 267 (297). Anal. Calcd. for C_13_H_14_N_6_: C 61.34; H 5.51; N 33.03. Found: C 60.92; H 5.58; N 33.02%.

### 3.3. Synthesis of the Complexes

#### 3.3.1. Synthesis of [Co{1,3-bis((1,2,4-triazol-1-ylmethyl)benzene-N,N}(H_2_O)_2_]Cl_2_ (**2**)

**L1** (0.991 mmol; 238.6 mg) was dissolved in acetone (6 mL), and CoCl_2_·6H_2_O (1.02 mmol; 241.8 mg) in acetone (8 mL) was added to this mixture. The resulting solution was stirred for 2 h at room temperature (r.t.). This mixture was centrifuged at 400 rpm for 8 min, washing with acetone, dichloromethane and ethyl ether and removing the liquid phase between each wash. Then, the solvent was evaporated to dryness to give a violet solid. Yield: 293.4 mg (90.6%). M.p.: > 400 °C. FTIR (KBr, cm^−1^): 3453, 3119, 1520, 1441, 1375, 1350, 1279, 1207, 1165, 1128, 1016, 988, 889, 735, 675, 650. Raman (cm^−1^): 1607, 1520, 1435, 1273, 1237, 1171, 1136, 1008, 753, 647, 501, 435. UV/Vis bands λ_max_, nm (ε, L mol^−1^ cm^−1^): 262 (784). Λ_*M*_ = 52.9 µS. Anal. Calcd. for C_24_H_28_Cl_2_CoN_12_O_2_: C 44.55; H 4.33; N 25.99. Found: C 45.00; H 4.21; N 25.96%. 

#### 3.3.2. Synthesis of [Co{2,6-bis((1,2,4-triazol-1-ylmetil)pyridine-N,N,N}(H_2_O)_3_] Cl_2_·H_2_O (**3**)

(**L2**) (1.0 mmol; 241.2 mg) was dissolved in acetone (9 mL), and CoCl_2_·6H_2_O (1.02 mmol; 241.8 mg) in acetone (7 mL) was added to this mixture. The resulting solution was stirred for 5 h at r.t. This mixture was centrifuged at 400 rpm for 8 min, washing with acetone, dichloromethane and ethyl ether and removing the liquid phase between each wash. Then, the solvent was evaporated to give a light-blue solid. Yield: 299 mg (67.4%). M.p.: > 400 °C. FTIR (KBr, cm^−1^): 3402, 3119, 1603, 1576, 1524, 1460, 1435, 1277, 1207, 1125, 1013, 986, 883, 766, 746, 677, 650. Raman (cm^−1^): 1430, 1348, 1125, 1014, 994, 593, 522, 342, 274. UV/Vis bands λ_max_, nm (ε, L mol^−1^ cm^−1^): 264 (3317). *Λ_M_* = 50.5 µS. Anal. Calcd. for C_11_H_19_Cl_2_CoN_7_O_4_: C 29.78; H 4.29; N 22.11. Found: C 29.69; H 3.94; N 22.07%. 

#### 3.3.3. Synthesis of [Co{3,5-bis((1,2,4-triazol-1-ylmethy)toluene-N,N}(H_2_O)_2_Cl_2_] (**4**)

1 (1.12 mmol; 241.8 mg) was dissolved in acetone (7 mL), and CoCl_2_·6H_2_O (1.02 mmol; 241.8 mg) in acetone (8 mL) was added to this mixture. The resulting solution was stirred for 3 h at r.t. This mixture was centrifuged at 400 rpm for 8 min, washing with acetone, dichloromethane and ethyl ether and removing the liquid phase between each wash. Then, the solvent was evaporated to give a blue solid. Yield: 296.3 mg (69.4%). M.p.: > 400 °C. FTIR (KBr, cm^−1^): 3111, 1609, 1522, 1437, 1352, 1279, 1206, 1128, 1016, 988, 881, 758, 675, 652. Raman (cm^−1^): 1605, 1520, 1464, 1434, 1358, 1282, 1127, 996, 759, 522, 303. UV/Vis bands λ_max_, nm (ε, L mol^−1^ cm^−1^): 268 (556). **Λ_*M*_** = 43.3 µS. Anal. Calcd. for C_13_H_18_Cl_2_CoN_6_O_2_: C 37.12; H 4.28; N 19.99. Found: C 37.11; H 4.28; N 20.06%. 

#### 3.3.4. Synthesis of [Cr {1,3-Bis(1,2,4-triazol-1ylmethyl)benzene-N,N}(H_2_O)Cl_3_]·2H_2_O (**5**)

**L1** (1.01 mmol; 241.9 mg) was dissolved in acetone (6 mL), and CrCl_3_·6H_2_O (1.0 mmol; 265.8 mg) in acetone (12 mL) was added to this mixture. The resulting solution was stirred for 2 h at r.t. This mixture was centrifuged at 400 rpm for 8 min, washing with acetone, dichloromethane and ethyl ether and removing the liquid phase between each wash. Then, the solvent was evaporated to give a light-green solid. Yield: 360.3 mg (79.8%). M.p.: > 400 °C. FTIR (KBr, cm^−1^): 3429, 1620, 1530, 1437, 1348, 1283, 1209, 1123, 1005, 889, 735, 673, 648. Raman (cm^−1^): 1609, 1528, 1430, 1362, 1278, 1237, 1172, 1123, 1000, 336, 268. UV/Vis bands λ_max_, nm (ε, L mol^−1^ cm^−1^): 260 (3672). Λ_*M*_ = 24.4 µS. Anal. Calcd. for C_12_H_18_Cl_3_CrN_6_O_3_: C 31.81; H 3.98; N 18.55. Found: C 31.39; H 4.24; N 18.39%. 

#### 3.3.5. Synthesis of [Cr{2,6-bis((1,2,4-triazol-1-ylmethyl)pyridine-N,N,N}(H_2_O)Cl_2_]Cl·5H_2_O (**6**)

**L2** (1.0 mmol; 241.7 mg) was dissolved in acetone (8 mL), and CrCl_3_·6H_2_O (1.01 mmol; 296.6 mg) in acetone (14 mL) was added to this mixture. The resulting solution was stirred for 2 h at r.t. This mixture was centrifuged at 400 rpm for 8 min, washing with acetone, dichloromethane and ethyl ether and removing the liquid phase between each wash. Then, the solvent was evaporated to give a light-green solid. Yield: 320 mg (63%). M.p.: > 400 °C. FTIR (KBr, cm^−1^): 3379, 3130, 1634, 1599, 1533, 1460, 1429, 1282, 1211, 1125, 1005, 887, 768, 673, 654. Raman (cm^−1^): 1425, 1366, 1121, 1084, 998, 587, 518, 338, 266. UV/Vis bands λ_max_, nm (ε, L mol^−1^ cm^−1^): 263 (7081). *Λ_M_* = 30.3 µS. Anal. Calcd. for C_11_H_23_Cl_3_CrN_7_O_6_: C 26.00; H 4.14; N 19.30. Found: C 26.05; H 4.04; N 19.19%. 

#### 3.3.6. Synthesis of [Cr{3,5-Bis(1,2,4-triazol-1-ylmethyl)toluene-N,N}(H_2_O)Cl_3_] 2H_2_O (**7**)

**1** (1.16 mmol; 293.8 mg) was dissolved in acetone (7 mL), and CrCl_3_·6H_2_O (1.02 mmol; 273.3 mg) in acetone (15 mL) was added to this mixture. The resulting solution was stirred for 2 h at r.t. This mixture was centrifuged at 400 rpm for 8 min, washing with acetone, dichloromethane and ethyl ether and removing the liquid phase between each wash. Then, the solvent was evaporated to give a light-green solid. Yield: 204.3 mg (43%). M.p.: > 400 °C. FTIR (KBr, cm^−1^): 3375, 3130, 1612, 1531, 1439, 1348, 1285, 1209, 1125, 1005, 887, 756, 671, 650. Raman (532 nm, cm^−1^): 1604, 1530, 1472, 1434, 1355, 1283, 1121, 997, 757, 518, 260. UV/Vis bands λ_max_, nm (ε, L mol^−1^ cm^−1^): 259 (2141). Λ_*M*_ = 27.4 µS. Anal. Calcd. for C_13_H_20_Cl_3_CrN_6_O_3_: C 33.42; H 4.29; N 17.99. Found: C 33.47; H 4.20; N 17.89%. 

### 3.4. Biological Studies

#### 3.4.1. Microorganisms and Mammalian Cells 

*Candida albicans* (ATCC, 10231), *C. tropicalis* (ATCC, 750), and *C. parasilopsis* (ATCC, 22019) were grown on Sabouraud Dextroseagar (OXOID Ltd., Basingstoke, Hampshire, UK) at 30 °C; *Staphylococcus aureus* (ATCC, 11632), *Escherichia coli* (ATCC, 10536) and *Salmonella typhimurium* (ATCC, 14028) were grown on nutrient agar (OXOID Ltd., Basingstoke, Hampshire, UK) at 37 °C. African green monkey kidney epithelial cells “Cercopithecusactiops” (Vero, CCL-81), donated by Dr. José Arteaga of the Universidad Industrial de Santander, were maintained in Dulbecco’s Modified Eagle’s Medium (DMEM, Life Technology, Carlsbad, CA, USA) supplemented with 5% inactivated fetal bovine serum (FBSi, Life Technology, Carlsbad, CA, USA) at 37 °C, 5% CO_2_ and 95% humidity. 

#### 3.4.2. Evaluation of the Antimicrobial Activity

Experiments were performed using the broth microdilution method according to the Clinical and Laboratory Standards Institute (CLSI) M27A-3 and M07-A10 protocols. Dilutions of each compound under study were performed in RPMI 1640 medium (Gibco, Life Technology, Carlsbad, CA, USA) supplemented with (3-(*N*-morpholino)propanesulfonic acid) (MOPS, Sigma-Aldrich) and Mueller Hinton broth (OXOID, Basingtoke, Hampshire, England) to concentrations of 2000–1.95 μg mL^−1^ in a 96-well plate. Untreated controls were similarly evaluated (itraconazole and amphotericin B on fungus and gentamicin and ampicillin on bacteria were purchased from Sigma-Aldrich (St. Louis, MI, USA). An inoculum containing between 2500 and 5000 cells/mL of each strain of Candida and 10^5^ cells/mL of bacteria was added (100 µL) to each well of the plate, and the plate was incubated at 37 °C for 48 h. The MIC was visually determined as the lowest concentration capable of inhibiting the growth of the fungus. Subsequently, the minimum fungicidal and bactericidal concentrations were determined after broth microdilution tests by sub-culturing a sample from the negative wells on the surface of Sabouraud dextroseand Mueller Hinton agar to determine the number of surviving cells after 24 h of incubation, and this value is expressed as CFU mL^−1^. The fungicidal and bactericidal endpoints were established as the lowest concentration that kills 98–99% of the final inoculum.

#### 3.4.3. Cytotoxicity Test on Mammalian Cells 

Vero cells (3 × 10^5^ cells/mL) were added to 96-well microplates and incubated for 24 h at 37 °C and 5% CO_2_until a monolayer formed. The cells were then treated with serial dilutions (1:3) of the compounds and reference drugs in concentrations of 300 μg mL^−1^ to 11.1 μg mL^−1^ for 72 h. The cell viability was evaluated using MTT (3-(4,5-dimethylthiazol-2-yl)-2,5-diphenyltetrazolium bromide, Sigma-Aldrich) tetrazolium salt at a concentration of 5 mg/mL. A spectrophotometric reading was performed on a microplate reader for measuring absorbance/ELISA (BIO-RAD) using a wavelength of 580 nm. All assays were performed in triplicate in independent experiments. The cytotoxic concentrations 50 and 90 (CC_50_ and CC_90_) were determined by sigmoidal regression using the statistical program XLFit5.

## 4. Conclusions

In summary, three ligands derived from triazole (one new) were synthesized and fully characterized. Additionally, six new complexes of Co(II) and Cr(III) were obtained, and they were characterized by spectroscopic, elemental and thermogravimetric techniques. The analyses and spectral data showed that complexes **3**–**7** had 1:1 (*M*:*L*) stoichiometries and octahedral geometries, while **2** had a 1:2 (*M*:*L*), and this was supported by DFT calculations. In vitro assays showed that the chromium complexes are more active against fungi than against bacterial strains, and they exhibited high anti-*C. tropicalis* activity. Interestingly, the observed activities appear to be related to the permanent dipole moment of each complex. These results can facilitate the design of new antibacterial and antifungal compounds. Studies to further elucidate their structure-activity relationship are in progress.

## Figures and Tables

**Table 1:** Electrical conductivity for **2**–**7** and starting salts.

Compound	Conductivity (Ω^−1^ cm^2^ mol^−1^)	Type of Electrolyte (In Solution)
CoCl_2_·6H_2_O	48.0	2:1
**2**	52.9	2:1
**3**	50.5	2:1
**4**	43.3	No electrolyte
CrCl_3_·6H_2_O	40.2	1:1
**5**	24.4	No electrolyte
**6**	30.3	1:1
**7**	27.4	No electrolyte

**Table 2:** Some bands for the starting salts, ligands and complexes 2–7 observed in their UV/Vis spectra.

	Transition (nm) (ε(M^−1^ cm^−1^))			
	UV		*Vis*	
Compound	*ν*_1_	*ν*_2_	*ν*_1_	*ν*_2_
**L1**	263(266)			
**2**	262(784)		615(66)	678(116)
**5**	260(3672)			
**L2**	264(4423)			
**3**	264(3317)		615(41)	677(79)
**6**	263(7081)			
**1**	267(297)	276(236)		
**4**	268(556)	276(478)	615(7)	675(26)
**7**	259(2141)			
CoCl_2_·6H_2_O	238(73)	266(64)	614(41)	678(81)
CrCl_3_·6H_2_O	260(2764)			

**Table 3:** Antimicrobial and cytotoxic activities of ligands and complexes (MIC, CC_50_; μg mL^−1^).

Compound	*S. aureus* (MIC)	*S. typhimurium* (MIC)	*E. coli* (MIC)	*C. tropicalis* (MIC)	*C. albicans* (MIC)	*C. parasilopsis* (MIC)	*Vero Cells* (CC_50_ ± SD)
**L1**	>2000	1000	>2000	>2000	>2000	>2000	>300
**L2**	>2000	1000	>2000	>2000	>2000	>2000	>300
**1**	>2000	2000	>2000	>2000	>2000	>2000	>300
**2**	1000–2000	1000	>2000	125	125	500	277.07 ± 1.86
**3**	250–500	1000	1000	31.25	125	125	68.76 ± 0.97
**4**	250–500	1000	2000	62.50	125	125	193.63 ± 6.51
**5**	>2000	1000	2000	7.81	62.5	125	>300
**6**	>2000	>2000	>2000	7.81	62.5	62.5	>300
**7**	>2000	>2000	>2000	15.62	31.25	125	130.60 ± 3.57
Ampicillin	0.078–0.156						
Gentamicin		2.50	0.625				
Amphotericin B				0.065	0.31	0.23	
Itraconazole				2.0	0.78	500	25.22 ± 3.51

The results are expressed in μg mL^−1^ and are the average of three independent experiments.

## Supplementary Materials

The following are available online.

## References

[1] O’Neill J. (2016). Tackling Drug-Resistant Infections Globally: Final Report and Recommendations. https://amr-review.org/sites/default/files/160518_Final%20paper_with%20cover.pdf.

[2] Crump J.A., Ramadhani H.O., Morrissey A.B., Saganda W., Mwako M.S., Yang L.Y., Chow S.C., Morpeth S.C., Reyburn H., Njau B.N. (2011). Invasive bacterial and fungal infections among hospitalized HIV-infected and HIV-uninfected adults and adolescents in northern Tanzania. Clin. Infect. Dis..

[3] Richardson M.D., Warnock D.W. (2012). Fungal Infection: Diagnosis and Management.

[4] Browning D.F., Busby S.J. (2016). Local and Global Regulation of Transcription Initiation in Bacteria. Nat. Rev. Microbiol..

[5] Kontoyiannis D.P., Vaziri I., Hanna H.A., Boktour M., Thornby J., Hachem R., Bodey G.P., Raad I.I. (2001). Risk Factors for *Candida Tropicalis* Fungemia in Patients with Cancer. Clin. Infect. Dis..

[6] Kothavade R.J., Kura M.M., Valand A.G., Panthaki M.H. (2010). *Candida Tropicalis*: Its Prevalence, Pathogenicity and Increasing Resistance to Fluconazole. J. Med. Microbiol..

[7] Silva S., Negri M., Henriques M., Oliveira R., Williams D.W., Azeredo J. (2012). *Candida Glabrata*, *Candida Parapsilosis* and *Candida Tropicalis*: Biology, Epidemiology, Pathogenicity and Antifungal Resistance. FEMS Microbiol. Rev..

[8] Bihan C.L., Zahar J.R., Timsit J.F. (2017). *Staphylococcus aureus* Transmission in the Intensive Care Unit: The Potential Role of the Healthcare Worker Carriage. Ann. Infect..

[9] Price J.R., Cole K., Bexley A., Kostiou V., Eyre D.W., Golubchik T., Wilson D.J., Crook D.W., Walker A.S., Peto T.E. (2017). Transmission of *Staphylococcus aureus* between Health-Care Workers, the Environment, and Patients in an Intensive Care Unit: A Longitudinal Cohort Study Based on Whole-Genome Sequencing. Lancet Infect. Dis..

[10] Recker M., Laabei M., Toleman M.S., Reuter S., Saunderson R.B., Blane B., Török M.E., Ouadi K., Stevens E., Yokoyama M. (2017). Clonal Differences in *Staphylococcus aureus* bacteraemia-Associated Mortality. Nat. Microbiol..

[11] Pierce R., Lessler J., Popoola V.O., Milstone A.M. (2017). Meticillin-Resistant *Staphylococcus aureus* (MRSA) Acquisition Risk in an Endemic Neonatal Intensive Care Unit with an Active Surveillance Culture and Decolonization Programme. J. Hosp. Infect..

[12] Kathiravan M.K., Salake A.B., Chothe A.S., Dudhe P.B., Watode R.P., Mukta M.S., Gadhwe S. (2012). The Biology and Chemistry of Antifungal Agents: A Review. Bioorg. Med. Chem..

[13] Bendaha H., Yu L., Touzani R., Souane R., Giaever G., Nislow C., Boone C., El Kadiri S., Brown G.W., Bellaoui M. (2011). New azole antifungal agents with novel modes of action: Synthesis and biological studies of new tridentate ligands based on pyrazole and triazole. Eur. J. Med Chem..

[14] Shrinivas D.J., Sheshagiri R.D., Uttam A.M., Tejraj A., Venkatrao H.K., Andanappa K.G. (2014). Enoyl ACP Reductase as Effective Target for the Synthesized Novel Antitubercular Drugs: A-State-of-the-Art. Mini Rev. Med. Chem..

[15] Singh K., Singh D.P., Barwa M.S., Tyagi P., Mirza Y. (2006). Antibacterial Co(II), Ni(II), Cu(II) and Zn(II) Complexes of Schiff bases Derived from Fluorobenzaldehyde and Triazoles. J. Enzyme Inhib. Med. Chem..

[16] Holla B.S., Poojary K.N., Rao B.S., Shivananda M.K. (2002). New bis-aminomercaptotriazoles and bis-triazolothiadiazoles as possible anticancer agents. Eur. J. Med. Chem..

[17] El-Gammal O.A., Bekheit M.M., Tahoon M. (2015). Synthesis, Characterization and Biological Activity of 2-Acetylpyridine-α-Naphthoxyacetylhydrazone Its Metal Complexes. Spectrochim. Acta A Mol. Biomol. Spectrosc..

[18] Hurtado J., Ibarra L., Yepes D., García-Huertas P., Macías M.A., Triana-Chavez O., Nagles E., Suescun L., Muñoz-Castro A. (2017). Synthesis, crystal structure, catalytic and anti-*Trypanosomacruzi* activity of a new chromium(III) complex containing bis(3,5-dimethylpyrazol-1-yl)methane. J. Mol. Struct..

[19] Alaghaz A.-N.M.A., Ammar R.A. (2010). New Dimeric Cyclodiphosph(V)Azane Complexes of Cr(III), Co(II), Ni(II), Cu(II), and Zn(II): Preparation, Characterization and Biological Activity Studies. Eur. J. Med. Chem..

[20] Tarafder M.T.H., Ali M.A., Wee D.J., Azahari K., Silong S., Crouse K.A. (2000). Complexes of a Tridentate ONS Schiff Base. Synthesis and Biological Properties. Transit. Met. Chem..

[21] Mohamed G.G., Zayed M.A., Abdallah S.M. (2010). Metal Complexes of a Novel Schiff Base Derived from Sulphametrole and Varelaldehyde. Synthesis, Spectral, Thermal Characterization and Biological Activity. J. Mol. Struct..

[22] Emara A.A.A. (2010). Structural, Spectral and Biological Studies of Binuclear Tetradentate Metal Complexes of N_3_O Schiff Base Ligand Synthesized from 4,6-Diacetylresorcinol and Diethylenetriamine. Spectrochim. Acta A Mol. Biomol. Spectrosc..

[23] Kljun J., Scott A.J., LanišnikRižner T., Keiser J., Turel I. (2014). Synthesis and Biological Evaluation of Organoruthenium Complexes with Azole Antifungal Agents. First Crystal Structure of a Tioconazole Metal Complex. Organometallics.

[24] Bello-Vieda N.J., Pastrana H.F., Garavito M.F., Ávila A.G., Celis A.M., Muñoz-Castro A., Restrepo S., Hurtado J.J. (2018). Antibacterial Activities of Azole Complexes Combined with Silver Nanoparticles. Molecules.

[25] Pahontu E., Fala V., Gulea A., Poirier D., Tapcov V., Rosu T. (2013). Synthesis and Characterization of Some New Cu(II), Ni(II) and Zn(II) Complexes with Salicylidene Thiosemicarbazones: Antibacterial, Antifungal and in Vitro Antileukemia Activity. Molecules.

[26] Yousef T.A., Abu El-Reash G.M., Al-Jahdali M., El-Rakhawy E.-B.R. (2014). Synthesis, spectral characterization and biological evaluation of Mn(II), Co(II), Ni(II), Cu(II), Zn(II) and Cd(II) complexes with thiosemicarbazone ending by pyrazole and pyridyl rings. Spectrochim. Acta A Mol. Biomol. Spectrosc..

[27] Castillo K.F., Bello-Vieda N.J., Nuñez-Dallos N.G., Pastrana H.F., Celis A.M., Restrepo S., Hurtado J.J., Ávila A.G. (2016). Metal complex derivatives of azole: A study on their synthesis, characterization, and antibacterial and antifungal activities. J. Braz. Chem. Soc..

[28] Sumrra S.H., Suleman A., Chohan Z.H., Zafar M.N., Raza M.A., Iqbal T. (2017). Triazole Metal Based Complexes as Antibacterial/Antifungal Agents. Russ. J. General Chem..

[29] Singh K., Kumar Y., Puri P., Kumar M., Sharma C. (2012). Cobalt, Nickel, Copper and Zinc Complexes with 1,3-Diphenyl-1H-Pyrazole-4-Carboxaldehyde Schiff Bases: Antimicrobial, Spectroscopic, Thermal and Fluorescence Studies. Eur. J. Med. Chem..

[30] Sundaraganesan N., Kavitha E., Sebastian S., Cornard J.P., Martel M. (2009). Experimental FTIR, FT-IR (gas phase), FT-Raman and NMR spectra, hyperpolarizability studies and DFT calculations of 3,5-dimethylpyrazole. Spectrochim. Acta A Mol. Biomol. Spectrosc..

[31] Geary W.J. (1971). The Use of Conductivity Measurements in Organic Solvents for the Characterization of Coordination Compounds. Coord. Chem. Rev..

[32] Katritzky A., Ramsden C., Joule J., Zhdankin V., Katritzky A.R. (2010). Handbook of Heterocyclic Chemistry.

[33] Sandoval-Rojas A.P., Ibarra L., Cortés M.T., Macías M.A., Suescun L., Hurtado J.J. (2017). Synthesis and characterization of copper(II) complexes containing acetate and N,N-donor ligands, and their electrochemical behavior in dopamine detection. J. Electroanal. Chem..

[34] Larkin P.J. (2011). Infrared and Raman Spectroscopy: Principles and Spectral Interpretation.

[35] Socrates G. (2001). Infrared and Raman Characteristic Group Frequencies: Tables and Charts.

[36] Billes F., Ziegler I., Mikosch H. (2016). Vibrational Spectroscopic Study of Sodium-1,2,4-Triazole, an Important Intermediate Compound in the Synthesis of Several Active Substances. Spectrochim. Acta A Mol. Biomol. Spectrosc..

[37] Billes F., Endredi H., Keresztury G. (2000). Vibrational Spectroscopy of Triazoles and Tetrazole. J. Mol. Struct. THEOCHEM.

[38] Cotton F.A., Wilkinson G. (1999). Advanced Inorganic Chemistry.

[39] Heerding D.A., Chan G., DeWolf W.E., Fosberry A.P., Janson C.A., Jaworski D.D., McManus E., Miller W.H., Moore T.D., Payne D.J. (2001). 1,4-Disubstituted imidazoles are potential antibacterial agents functioning as inhibitors of enoyl acyl carrier protein reductase (FabI). Bioorg. Med. Chem. Lett..

[40] Kabbani A.T., Hammud H.H., Ghannoum A.M. (2007). Preparation and antibacterial activity of copper and cobalt complexes of 4-chloro-3-nitrobenzoate with a nitrogen donor ligand. Chem. Pharm. Bull. (Tokyo).

[41] Collin F., Karkare S., Maxwell A. (2011). Exploiting bacterial DNA gyrase as a drug target: Current state and perspectives. Appl. Microbiol. Biotechnol..

[42] McIlwain H. (1940). Pyridine-3-Sulphonic Acid and Its Amide as Inhibitors of Bacterial Growth. Br. J. Exp. Pathol..

[43] Schneider D., Parker C.D. (1982). Effect of Pyridines on Phenotypic Properties of *Bordetella Pertussis*. Infect. Immun..

[44] Bagihalli G.B., Avaji P.G., Patil S.A., Badami P.S. (2008). Synthesis, Spectral Characterization, *in Vitro* Antibacterial, Antifungal and Cytotoxic Activities of Co(II), Ni(II) and Cu(II) Complexes with 1,2,4-Triazole Schiff Bases. Eur. J. Med. Chem..

[45] Poyraz M., Sari M., Demirci F., Kosar M., Demirayak S., Büyükgüngör O. (2008). Synthesis, Crystal Structure and Biological Activity of 1-(1H-Benzoimidazol-2-Yl)-EthanoneThiosemicarbazone and Its Cobalt Complex. Polyhedron.

[46] Van den Bossche H., Willemsens G., Cools W., Marichal P., Lauwers W. (1983). Hypothesis on the molecular basis of the antifungal activity of N-substituted imidazoles and triazoles. Biochem. Soc. Trans..

[47] Herbrecht R. (2004). Voriconazole: Therapeutic review of a new azole antifungal. Expert Rev. Anti Infect. Ther..

[48] Chohan Z.H., Hanif M. (2010). Design, Synthesis, and Biological Properties of Triazole Derived Compounds and Their Transition Metal Complexes. J. Enzyme Inhib. Med. Chem..

[49] Chohan Z.H., Hanif M. (2013). Antibacterial and Antifungal Metal Based Triazole Schiff Bases. J. Enzyme Inhib. Med. Chem..

[50] Carcelli M., Mazza P., Pelizzi C., Zani F. (1995). Antimicrobial and Genotoxic Activity of 2,6-Diacetylpyridine Bis(Acylhydrazones) and Their Complexes with Some First Transition Series Metal Ions. X-Ray Crystal Structure of a Dinuclear Copper(II) Complex. J. Inorg. Biochem..

[51] Shreaz S., Sheikh R.A., Bhatia R., Neelofar K., Imran S., Hashmi A.A., Manzoor N., Basir S.F., Khan L.A. (2011). Antifungal Activity of α-Methyl Trans Cinnamaldehyde, Its Ligand and Metal Complexes: Promising Growth and Ergosterol Inhibitors. BioMetals.

[52] Bellú S., Hure E., Trapé M., Trossero C., Molina G., Drogo C., Williams P.A.M., Atria A.M., Muñoz Acevedo J.C., Zacchino S. (2005). Synthesis, Structure and Antifungal Properties of Co(II)–sulfathiazolate Complexes. Polyhedron.

[53] Rodríguez-Argüelles M.C., Cao R., García-Deibe A.M., Pelizzi C., Sanmartín-Matalobos J., Zani F. (2009). Antibacterial and Antifungal Activity of Metal(II) Complexes of Acylhydrazones of 3-Isatin and 3-(N-Methyl)Isatin. Polyhedron.

[54] Pea F., Lewis R.E. (2018). Overview of antifungal dosing in invasive candidiasis. J. Antimicrob. Chemother..

[55] TeVelde G., Bickelhaupt F.M., Baerends E.J., Fonseca Guerra C., van Gisbergen S.J.A., Snijders J.G., Ziegler T. (2001). Chemistry with ADF. J. Comput. Chem..

[56] Grimme S. (2006). Semiempirical GGA-type density functional constructed with a long-range dispersion correction. J. Comput. Chem..

[57] Lien E.J., Guo Z.R., Li R.L., Su C.T. (1982). Use of Dipole Moment as a Parameter in Drug-Receptor Interaction and Quantitative Structure-Activity Relationship Studies. J. Pharm. Sci..

[58] Vistoli G., Pedretti A. (2007). Molecular Fields to Assess Recognition Forces and Property Spaces. Comprehensive Medicinal Chemistry II.

[59] Díez-Barra E., Guerra J., López-Solera I., Merino S., Rodríguez-López J., Sánchez-Verdú P., Tejeda J. (2003). Novel Chiral and Achiral NCN Pincer Complexes Based on 1,3-Bis(1*H*-1,2,4-Triazol-1-Ylmethyl)Benzene. Organometallics.

[60] Kim E.Y., Song Y.J., Koo H.G., Lee J.H., Park H.M., Kim C., Kwon T.-H., Huh S., Kim S.-J., Kim Y. (2010). 1-D, 2-D and 3-D Coordination Polymers Assembled from PolynuclearCoII Units Based on the Isophthalate(-2) Ligand. Polyhedron.

