# Supplementary material for: Development of Antibacterial and Antifungal Triazole Chromium(III) and Cobalt(II) Complexes: Synthesis and Biological Activity Evaluations
Source: Molecules. 2018 Aug 13;23(8):2013. doi: 10.3390/molecules23082013 (PMC6222626; doi:10.3390/molecules23082013)
Supplement: Supplementary file 1 [file molecules-23-02013-s001.pdf]

Supplementary Materials

Development of Antibacterial and Antifungal Triazole Chromium(III) and Cobalt(II) Complexes: Synthesis and Biological Activity Evaluations

Ricardo A. Murcia[1], Sandra M. Leal[2,3], Martha V. Roa[2,3], Edgar Nagles[4], Alvaro Muñoz-Castro[5] and John J. Hurtado[1*]

[1]Departamento of Chemistry, Universidad de los Andes, Carrera 1 No. 18A-12, 111711, Bogotá-Colombia

[2]Programa de Bacteriología y Laboratorio Clínico, Facultad de Ciencias de la Salud, Universidad de Santander, Bucaramanga, Colombia

[3]Programa de Maestría en Investigación en Enfermedades Infecciosas, Facultad de Ciencias de la Salud, Universidad de Santander, Bucaramanga, Colombia

[4]Facultad de Ciencias Naturales y Matemáticas, Universidad de Ibagué, Carrera 22 Calle 67, 730001, Ibagué

[5]Grupo de Química Inorgánica y Materiales Moleculares, Universidad Autonoma de Chile, El Llano Subercaseaux 2801, Santiago, Chile.

*Corresponding author.
E-mail address: jj.hurtado@uniandes.edu.co (J. Hurtado), Tel.: +57-1-339-4949 (ext. 3468)
ORCID ID: 0000-0002-0511-9719

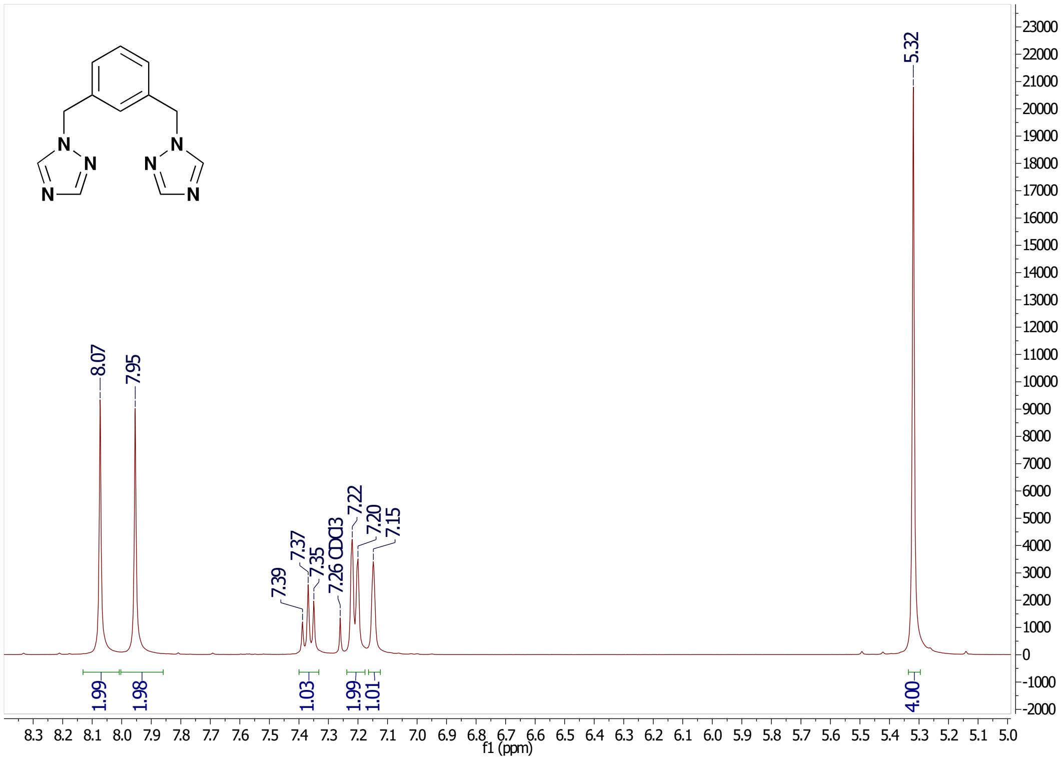

Figure S1. ^{1}H RMN spectrum of (L**1**). $CDCl_3$

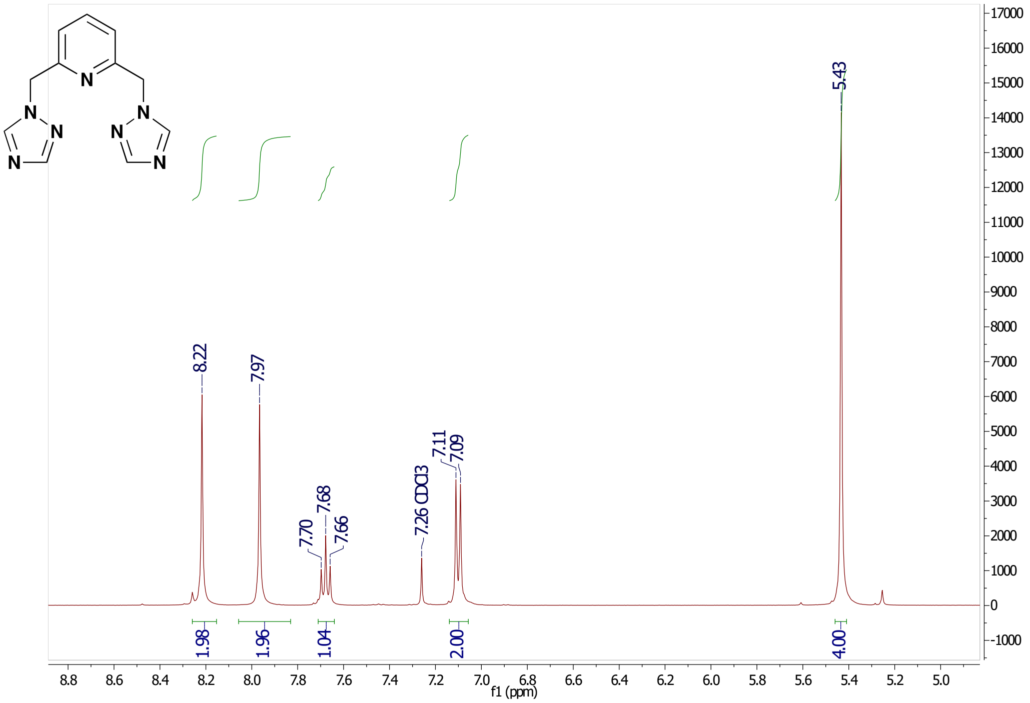

Figure S2. ^{1}H RMN spectrum of (**L2**). $CDCl_3$

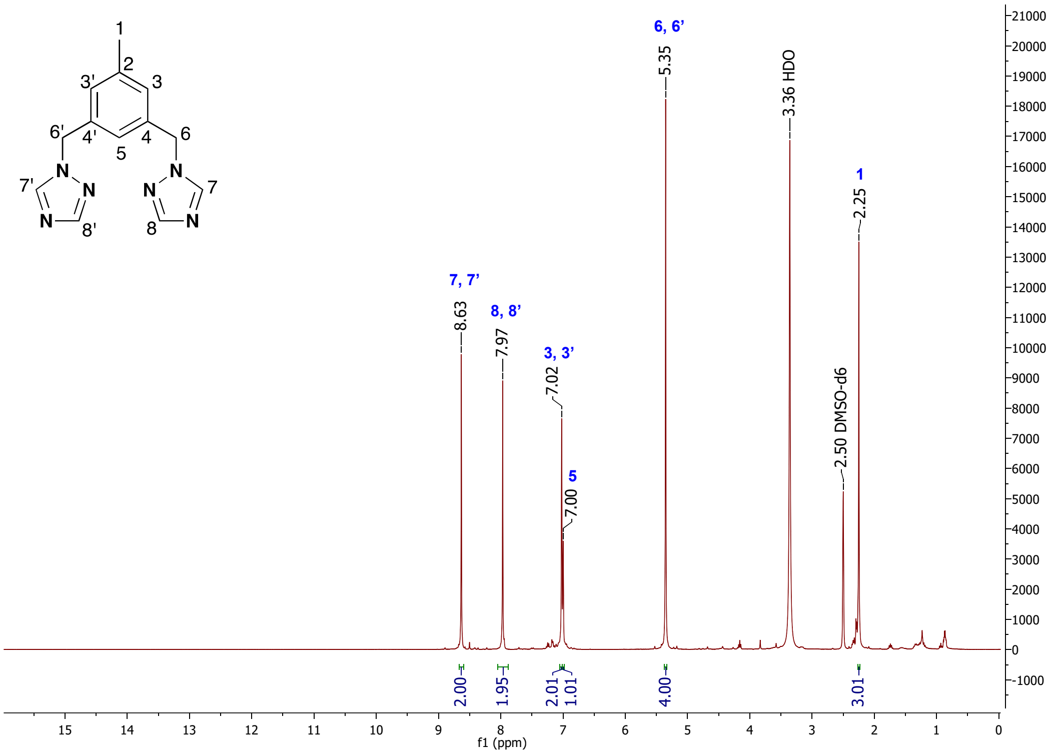

Figure S3. ^{1}H RMN spectrum of (**1**).

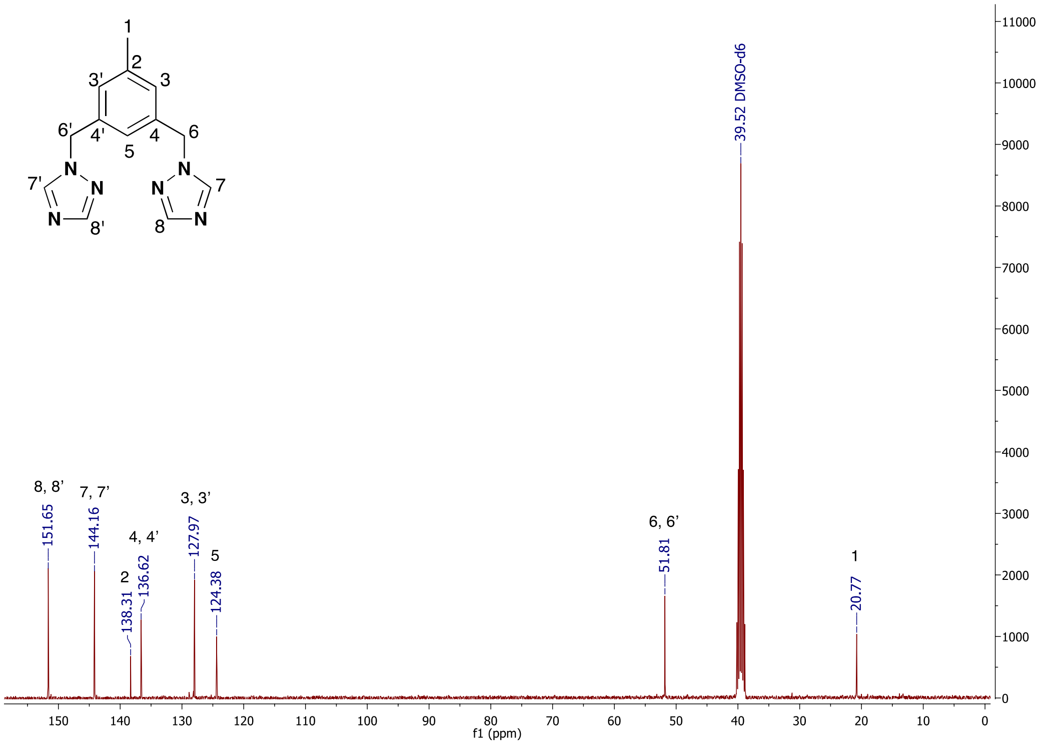

Figure S4. ^{13}C RMN spectrum of (**1**).

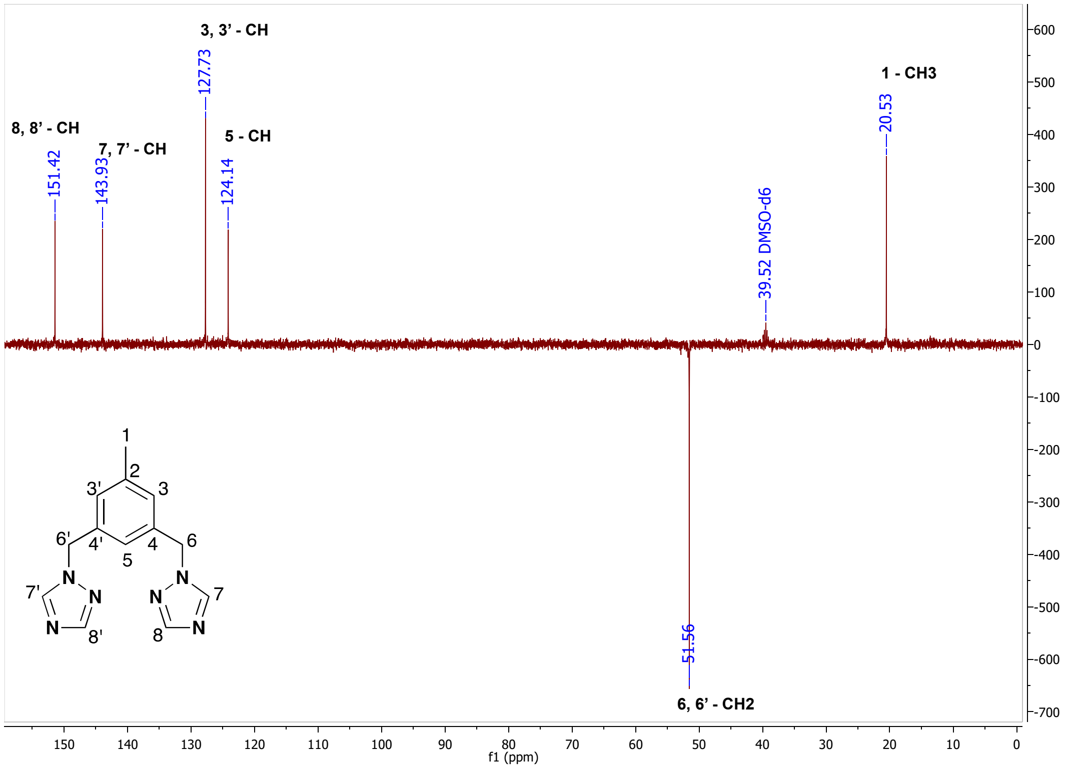

Figure S5. DEPT 135 spectrum of (**1**).

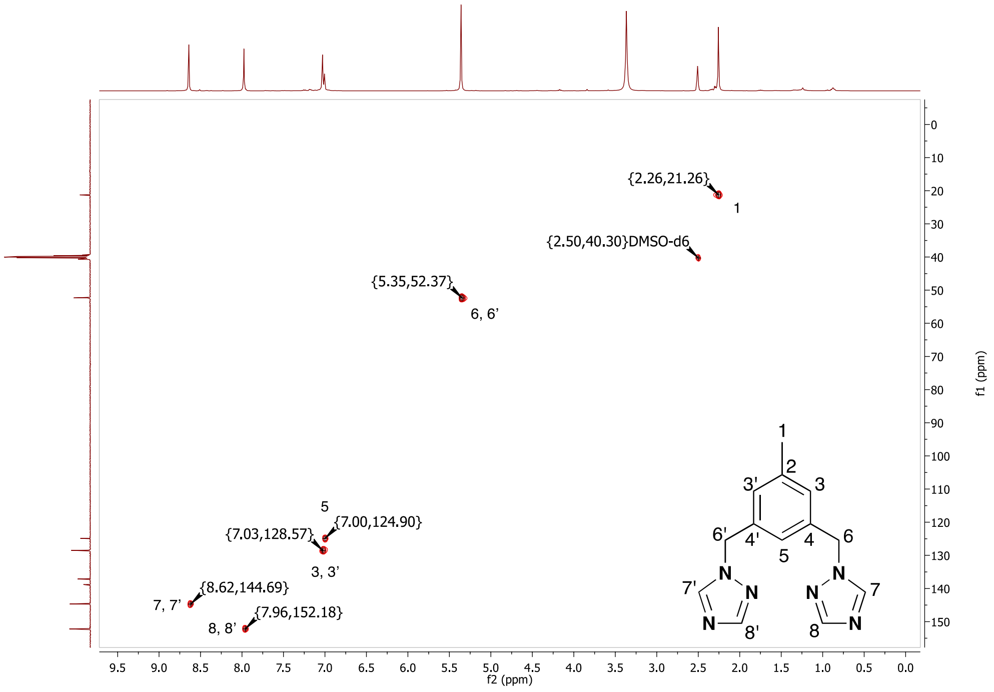

Figure S6. HSQC spectrum of (**1**).

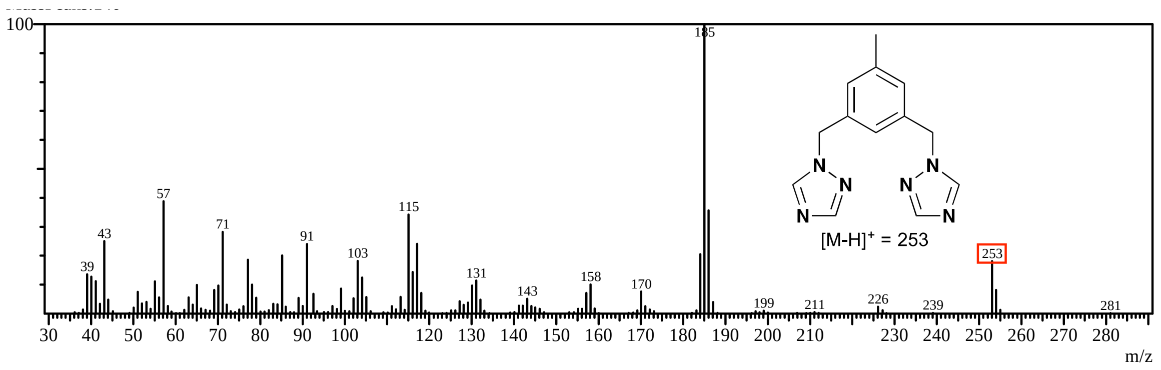

Figure S7. Mass spectrum of (**1**).

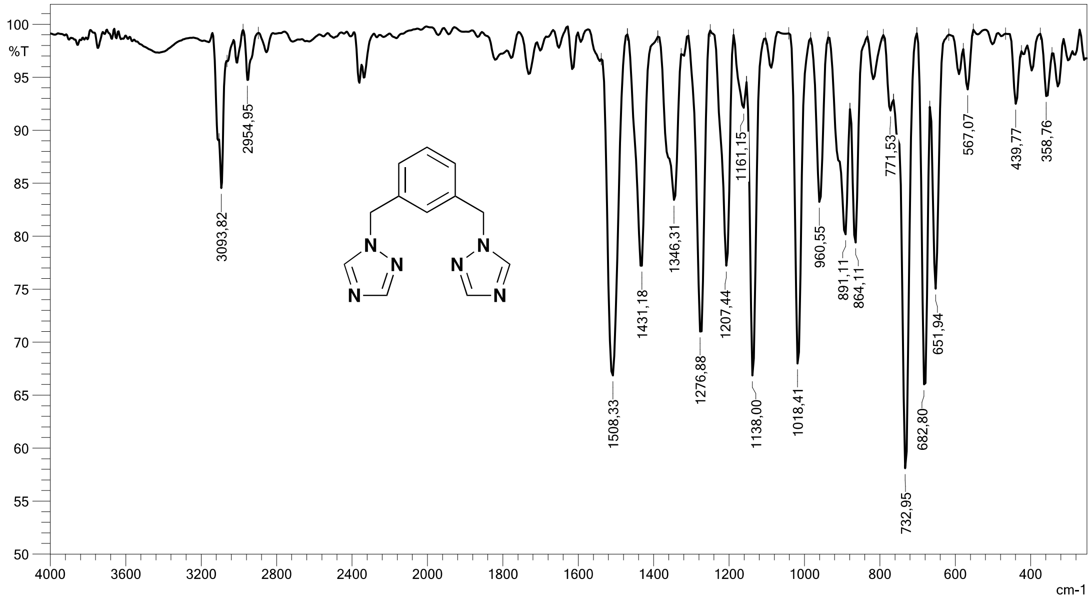

Figure S8. IR spectrum of (**L1**).

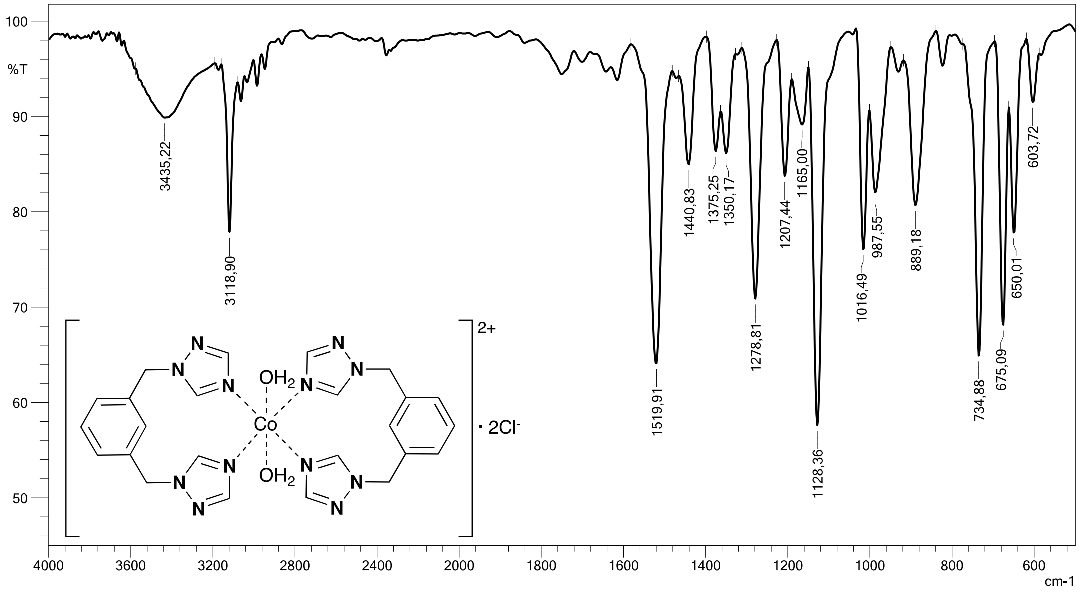

Figure S9. IR spectrum of (**2**).

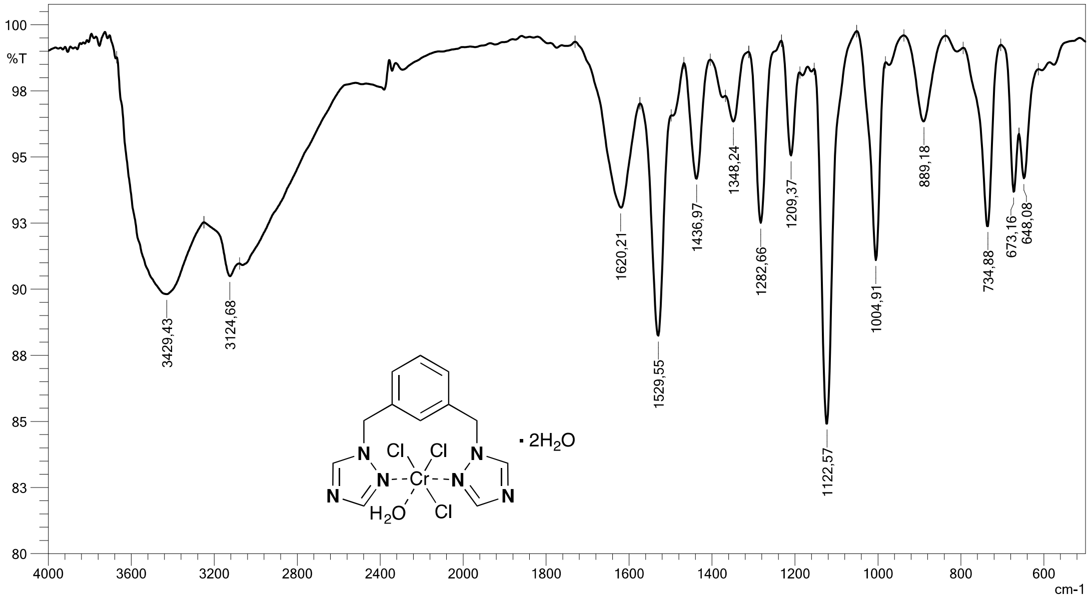

Figure S9. IR spectrum of (**5**).

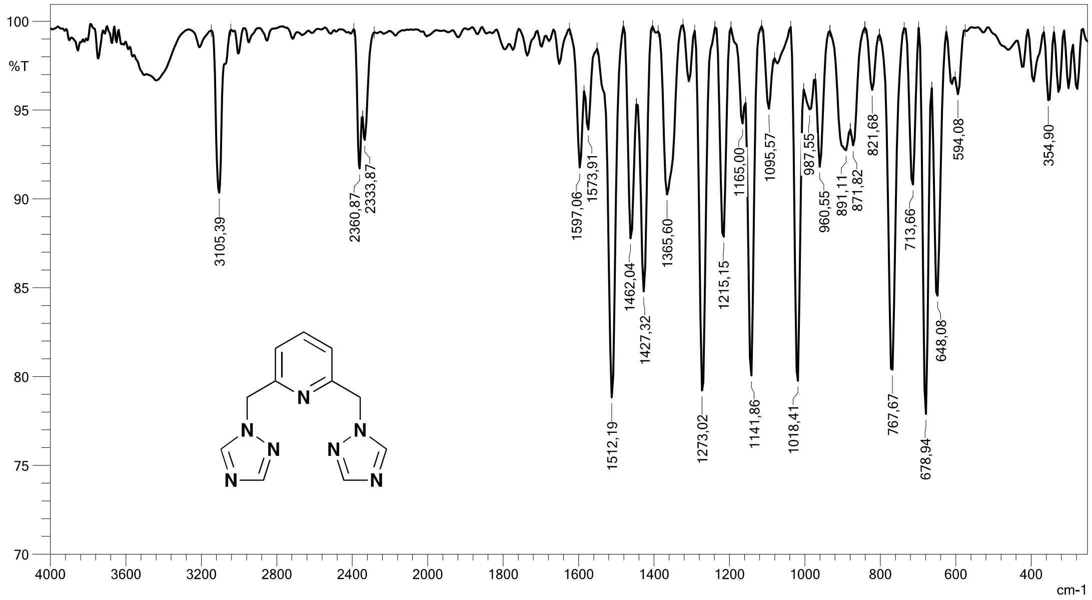

Figure S10. IR spectrum of (**L2**).

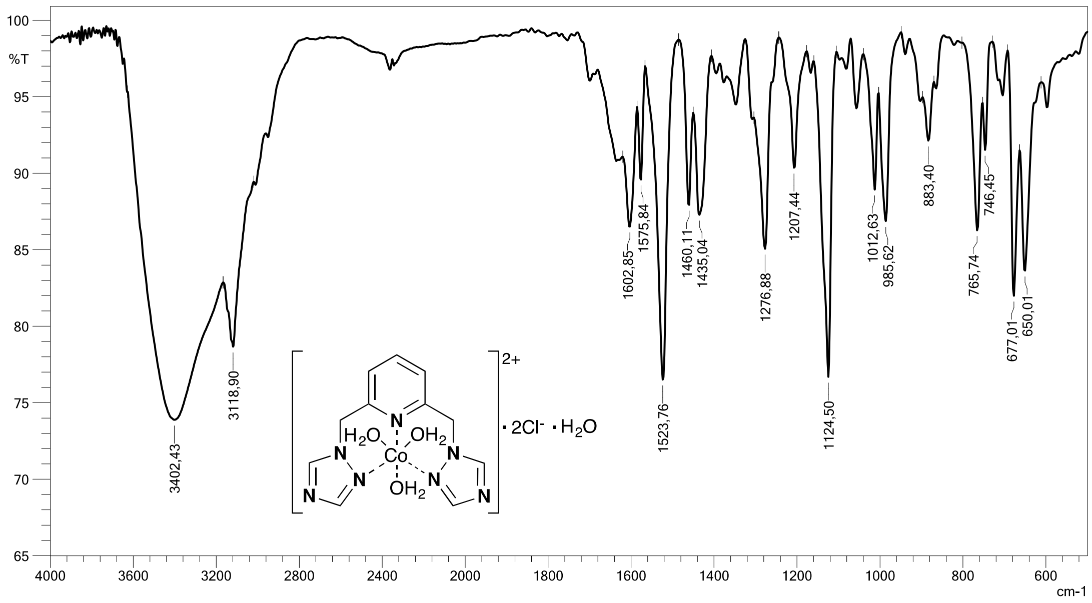

Figure S11. IR spectrum of (**3**).

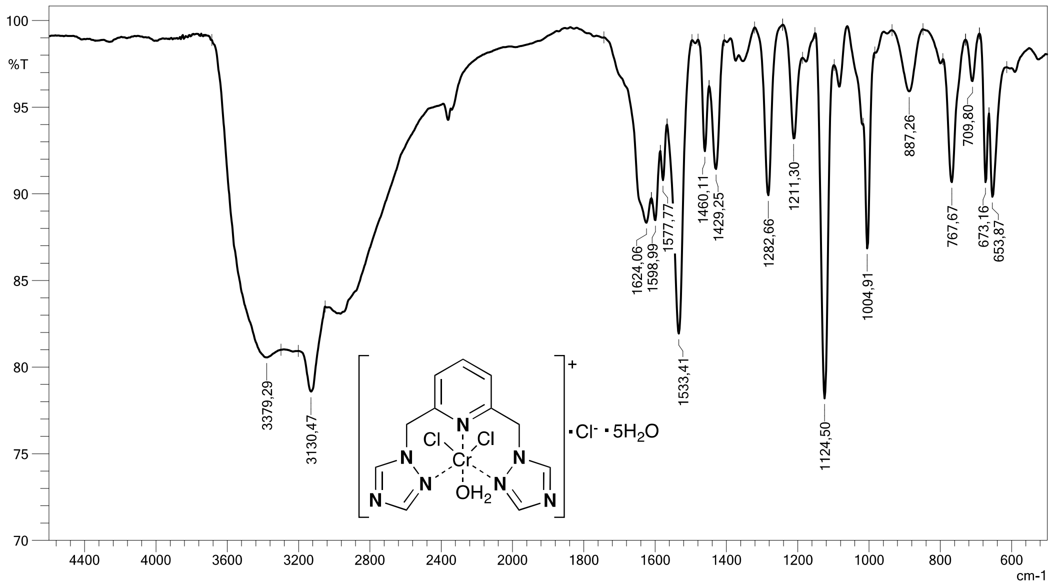

Figure S12. IR spectrum of (**6**).

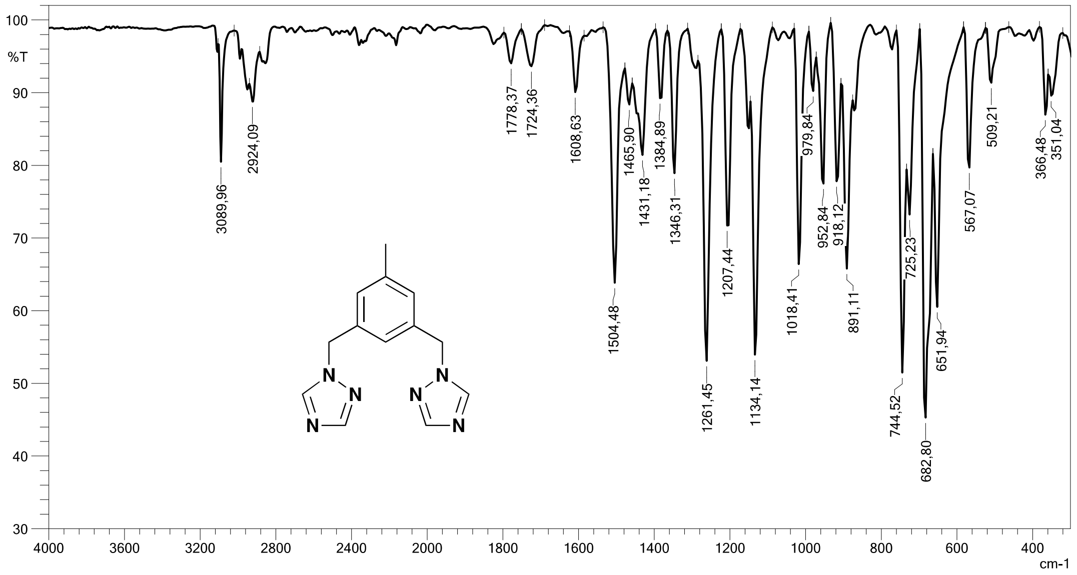

Figure S13. IR spectrum of (**1**).

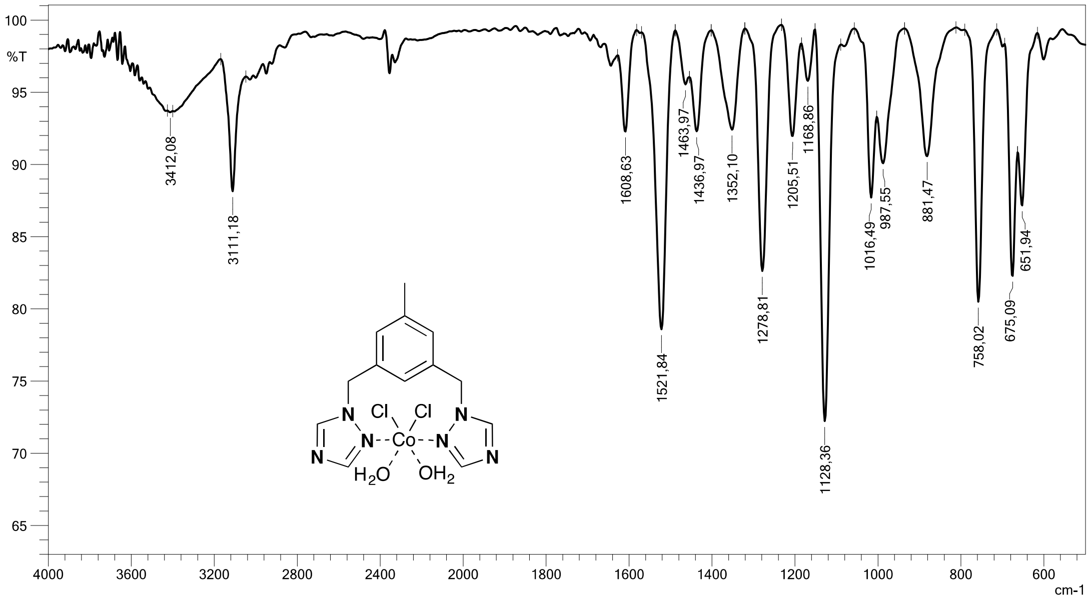

Figure S14. IR spectrum of (**4**).

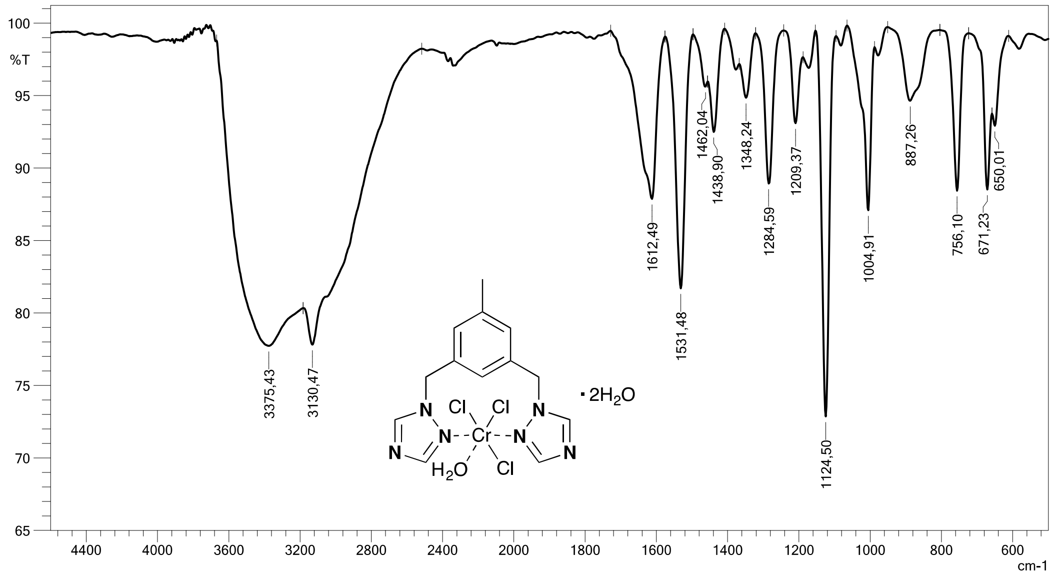

Figure S15. IR spectrum of (**7**).

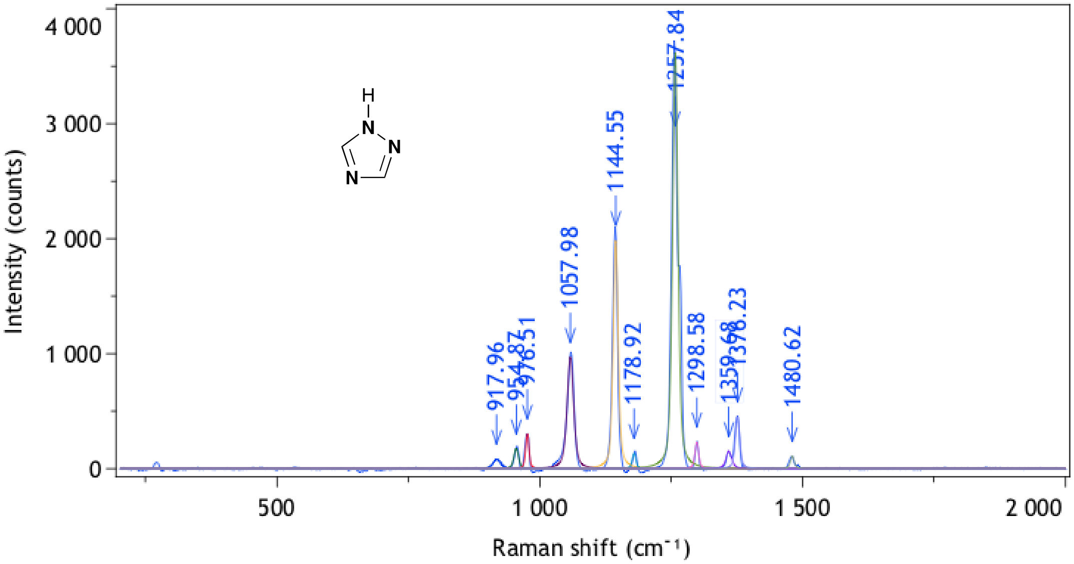

Figure S16. Raman spectrum of 1*H*-1,2,4-triazole.

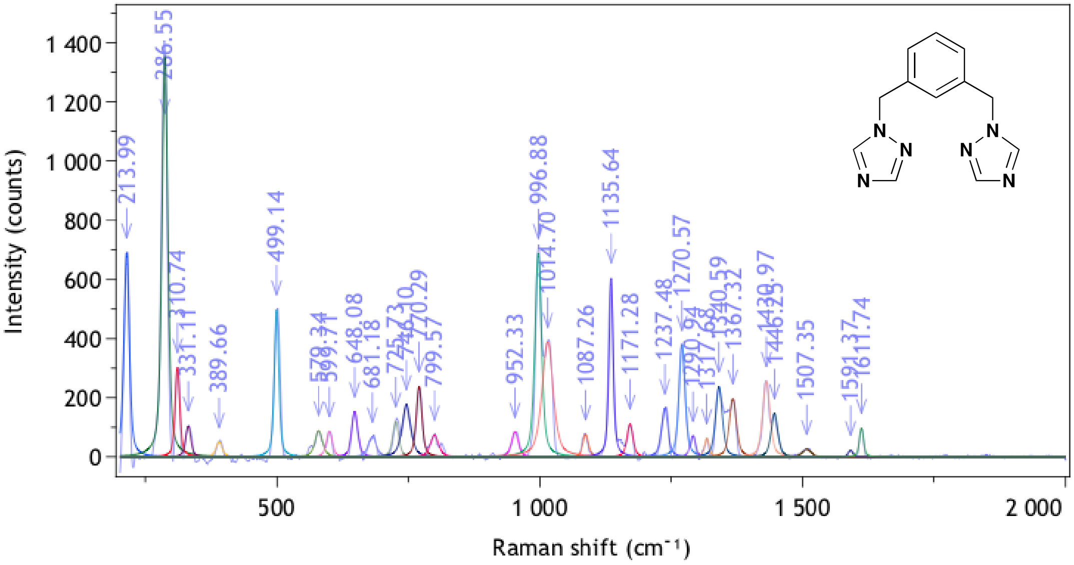

Figure S17. Raman spectrum of (**L1**).

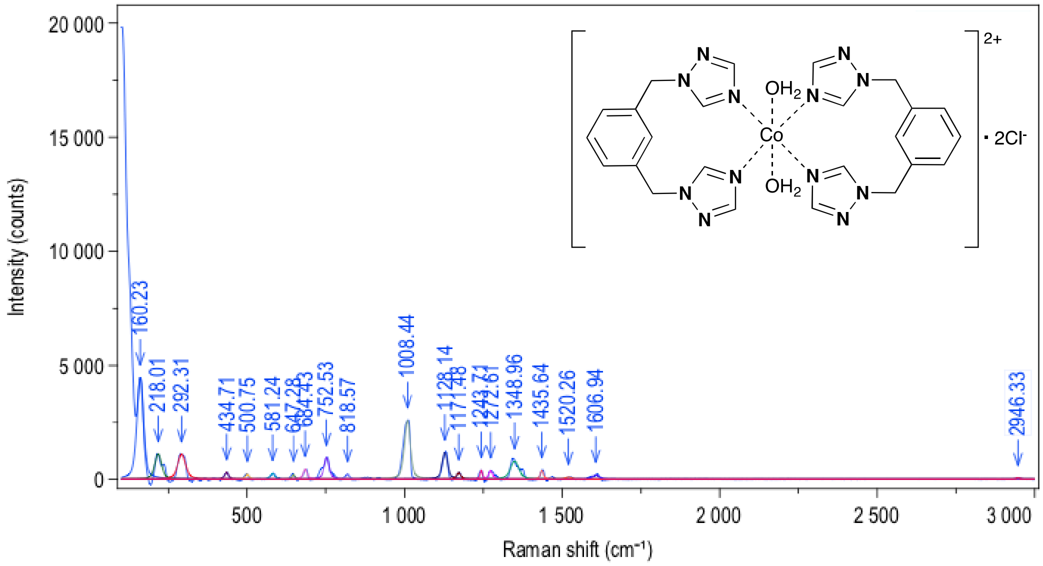

Figure S18. Raman spectrum of (**2**).

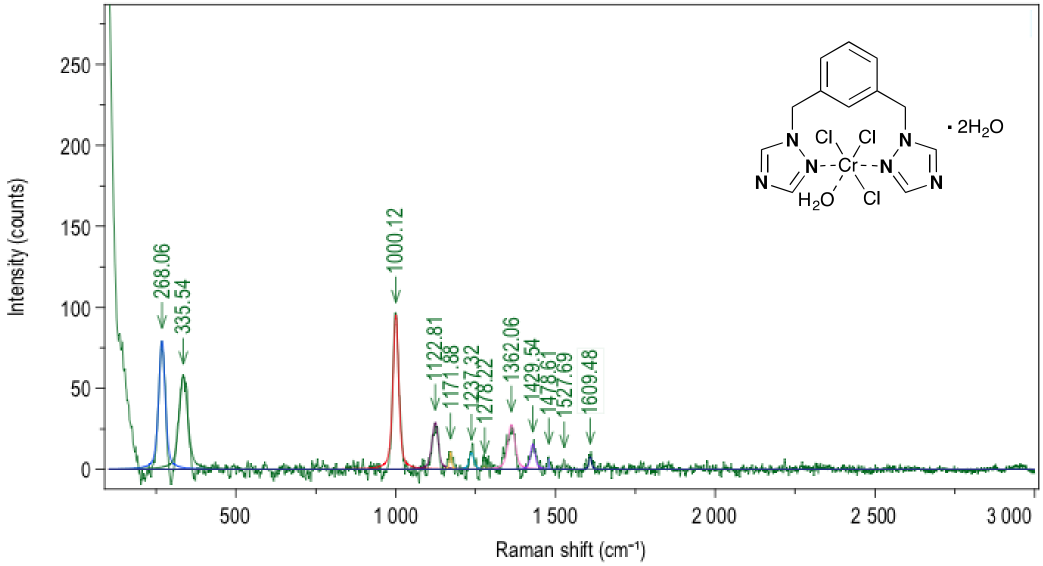

Figure S19. Raman spectrum of (**5**).

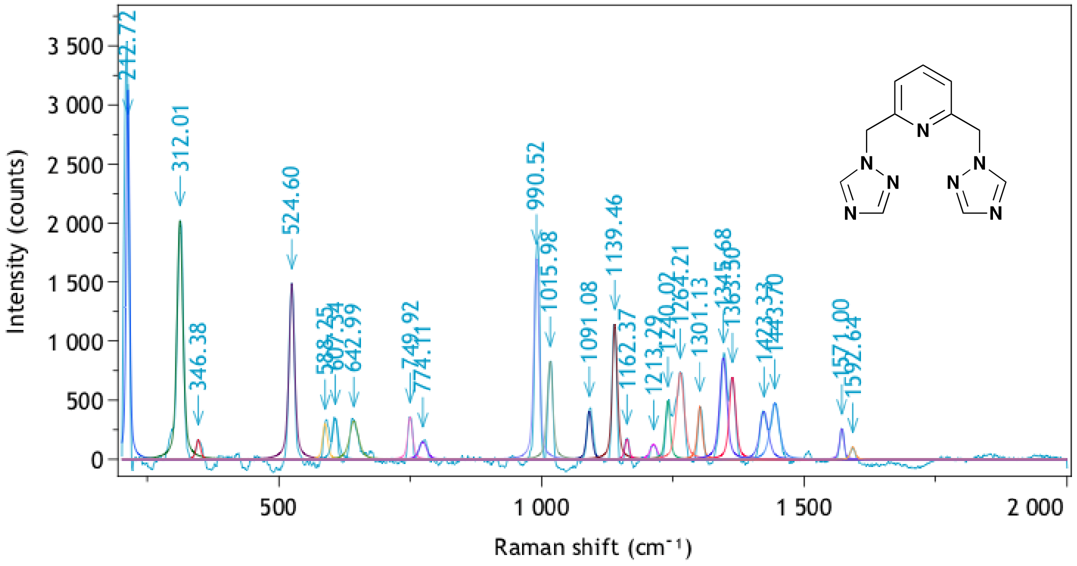

Figure S20. Raman spectrum of (**L2**).

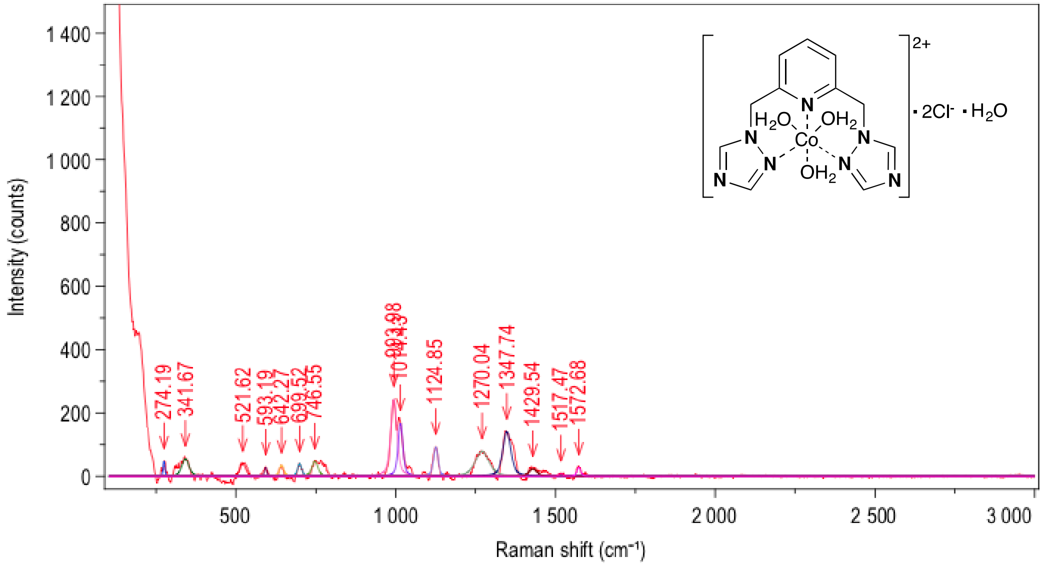

Figure S21. Raman spectrum of (**3**).

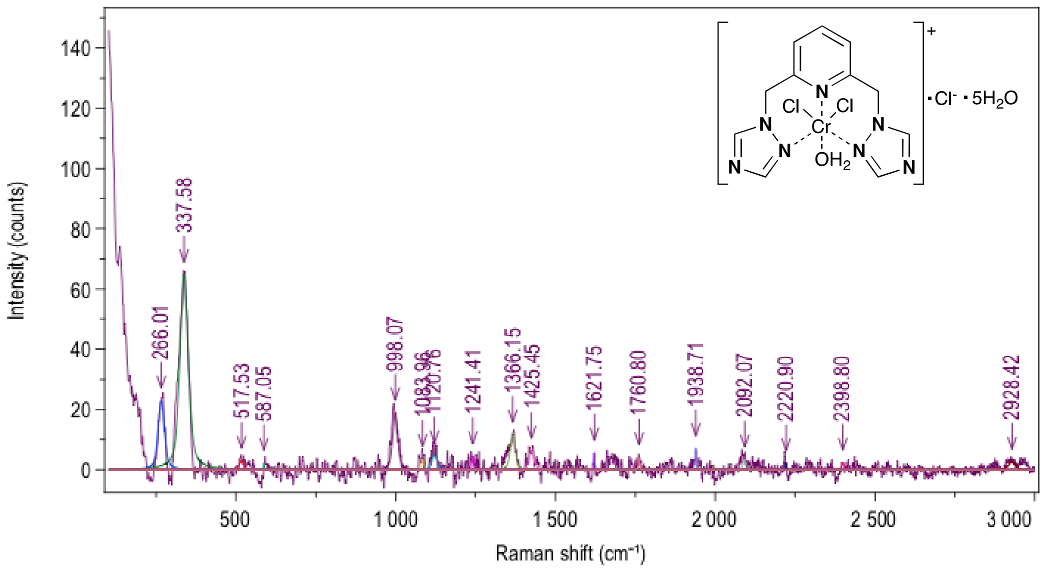

Figure S22. Raman spectrum of (**6**).

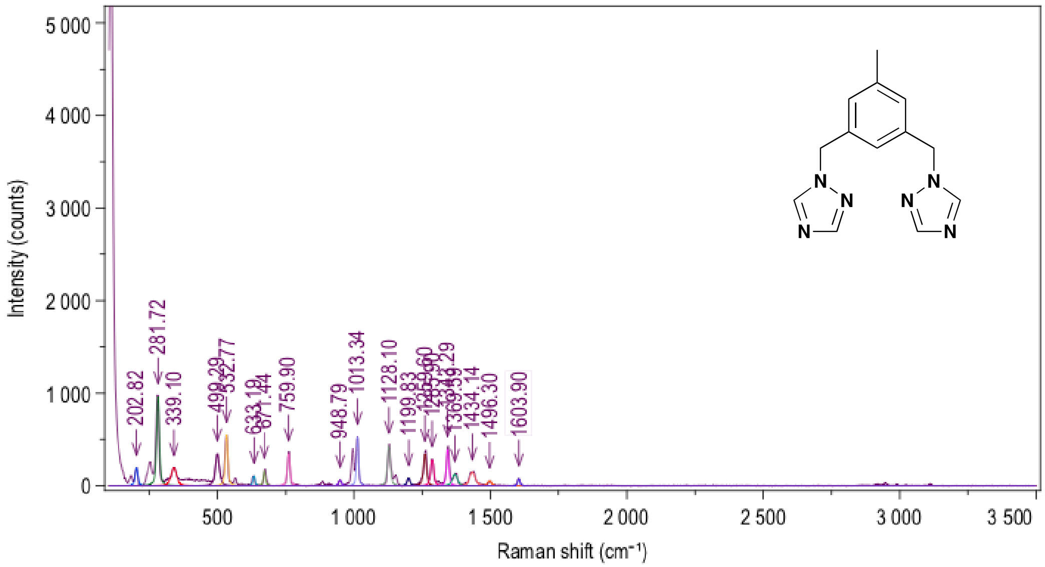

Figure S23. Raman spectrum of (**1**).

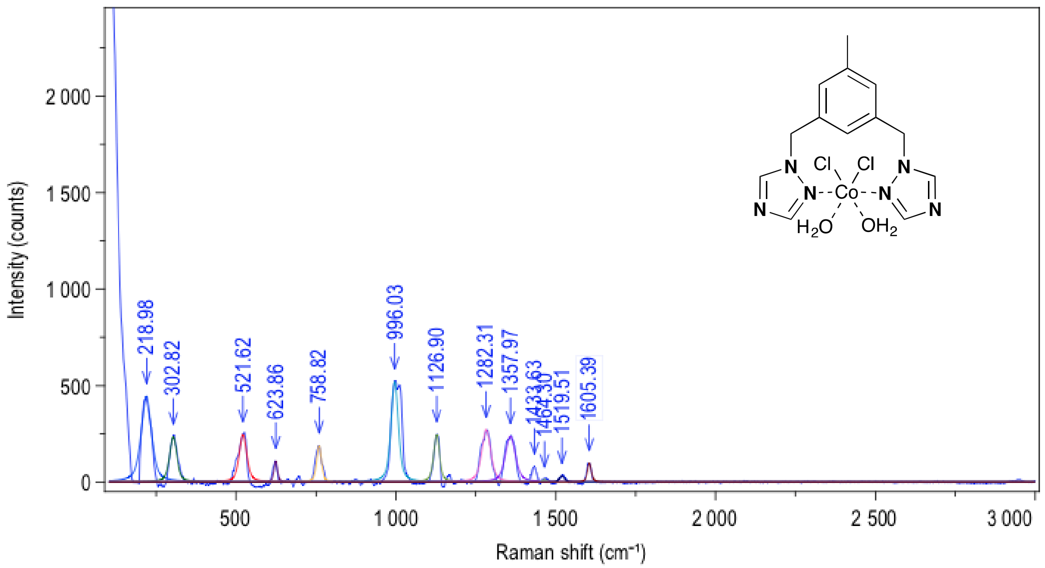

Figure S24. Raman spectrum of (**4**).

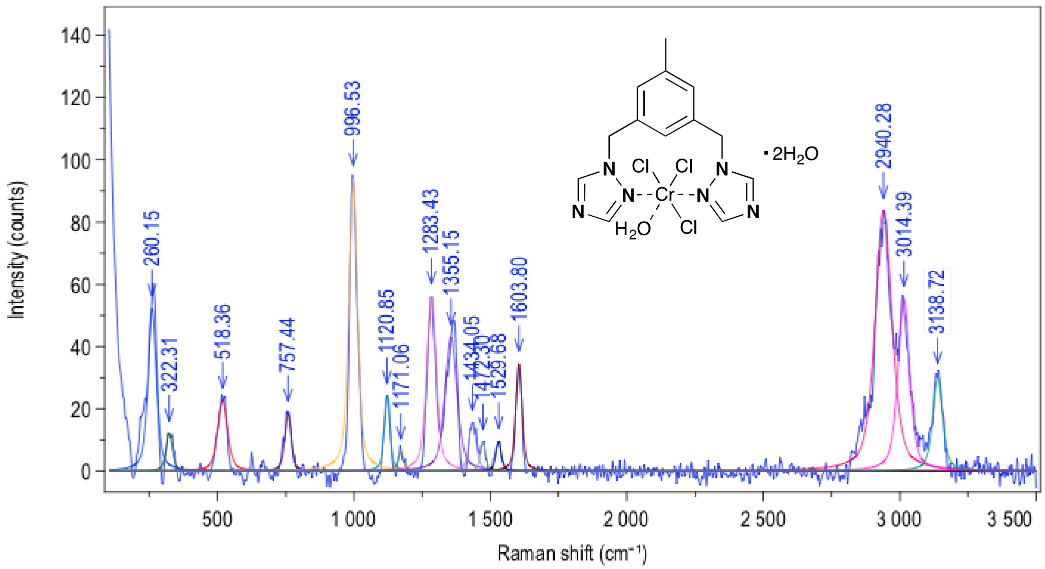

Figure S25. Raman spectrum of (**7**).

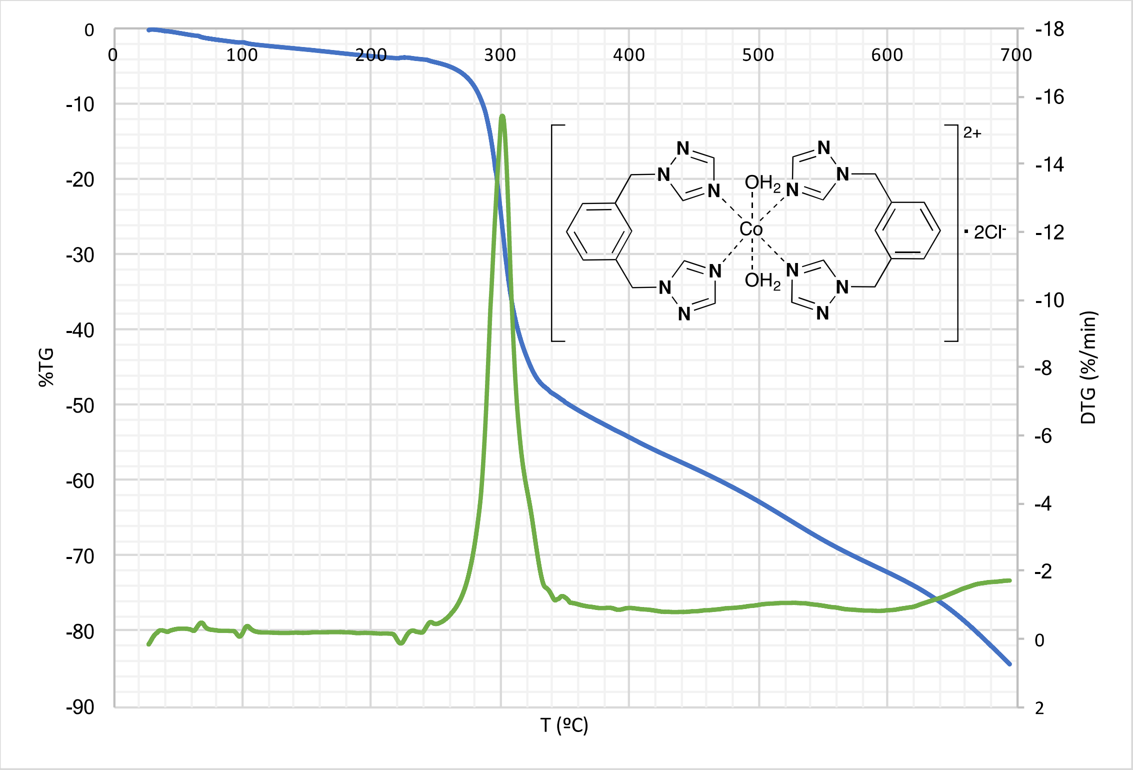

Figure S26. TGA and DTG of (**2**).

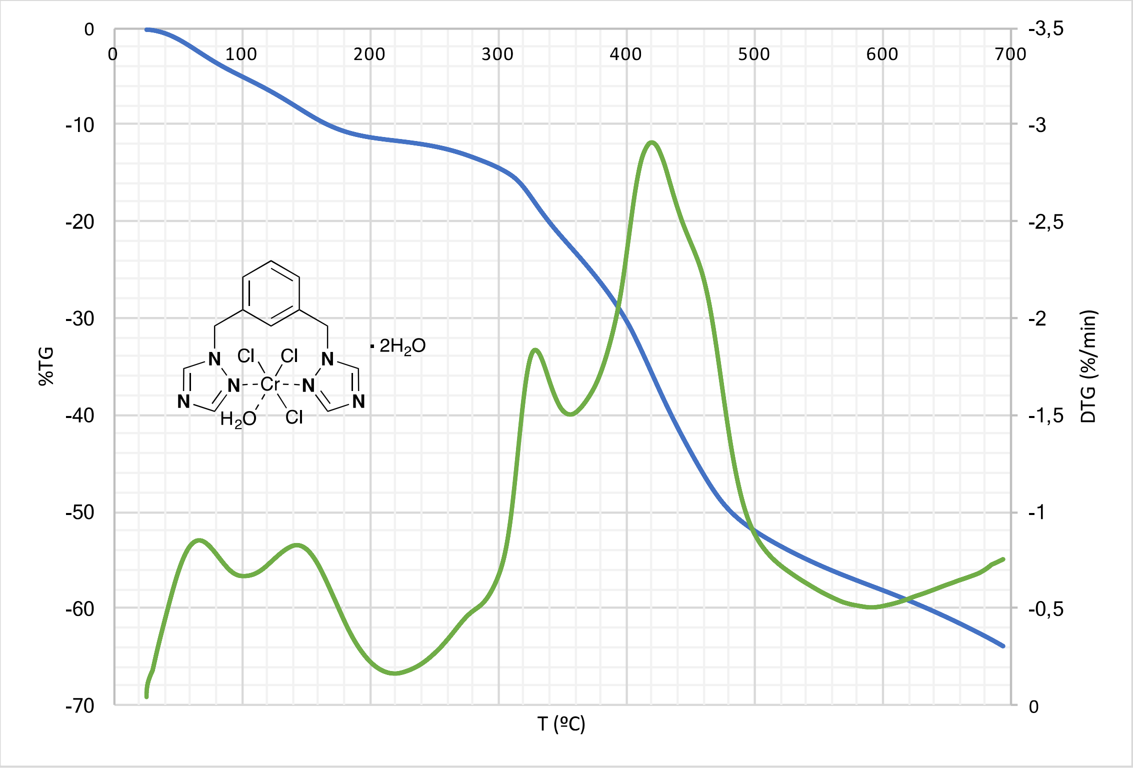

Figure S27. TGA and DTG of (**5**).

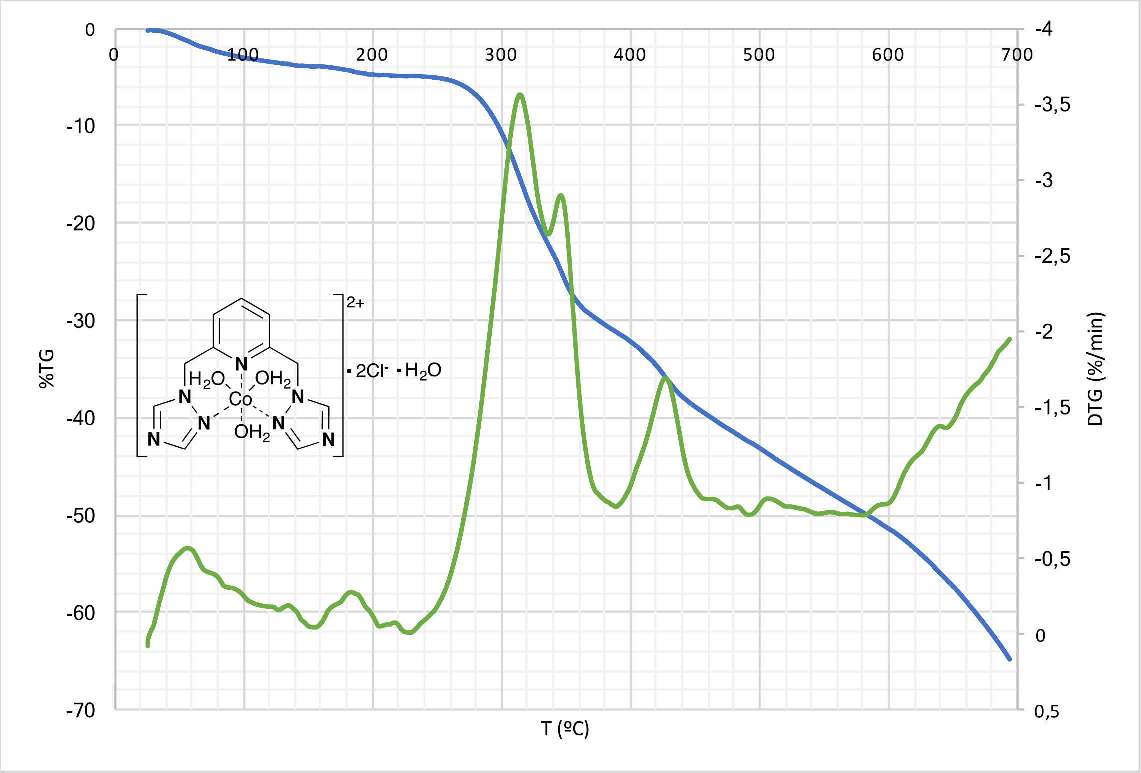

Figure S28. TGA and DTG of (**3**).

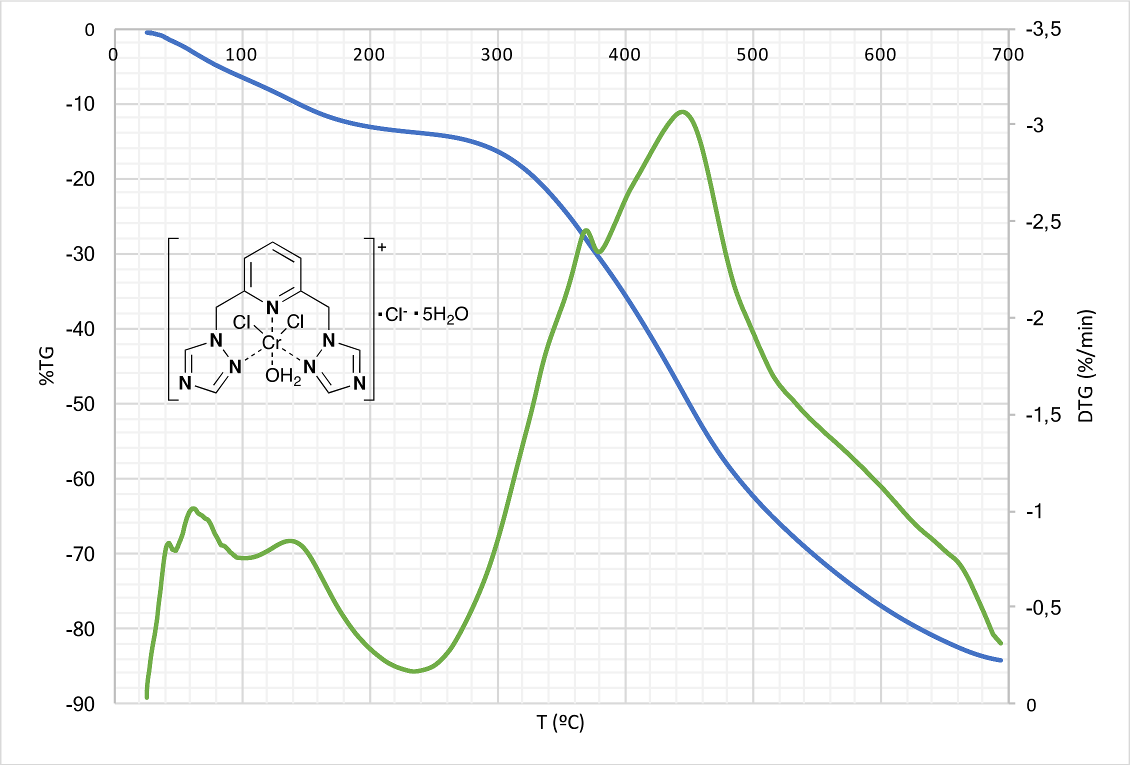

Figure S29. TGA and DTG of (**6**).

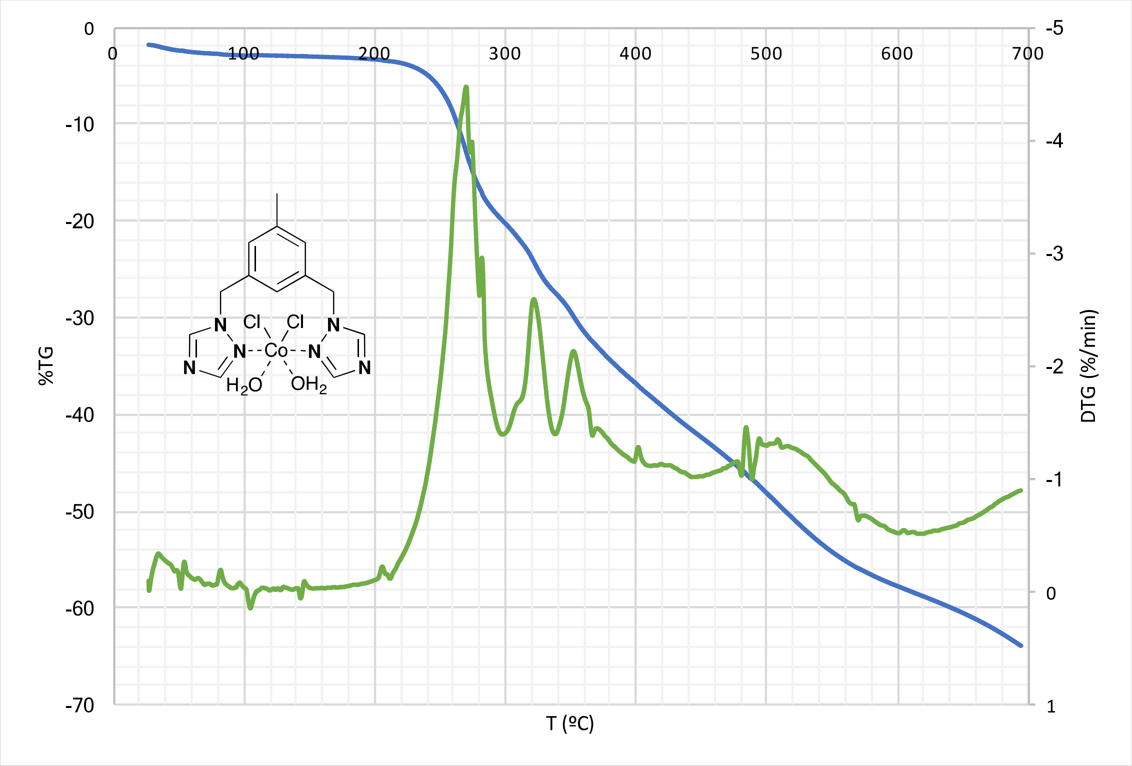

Figure S30. TGA and DTG of (**4**).

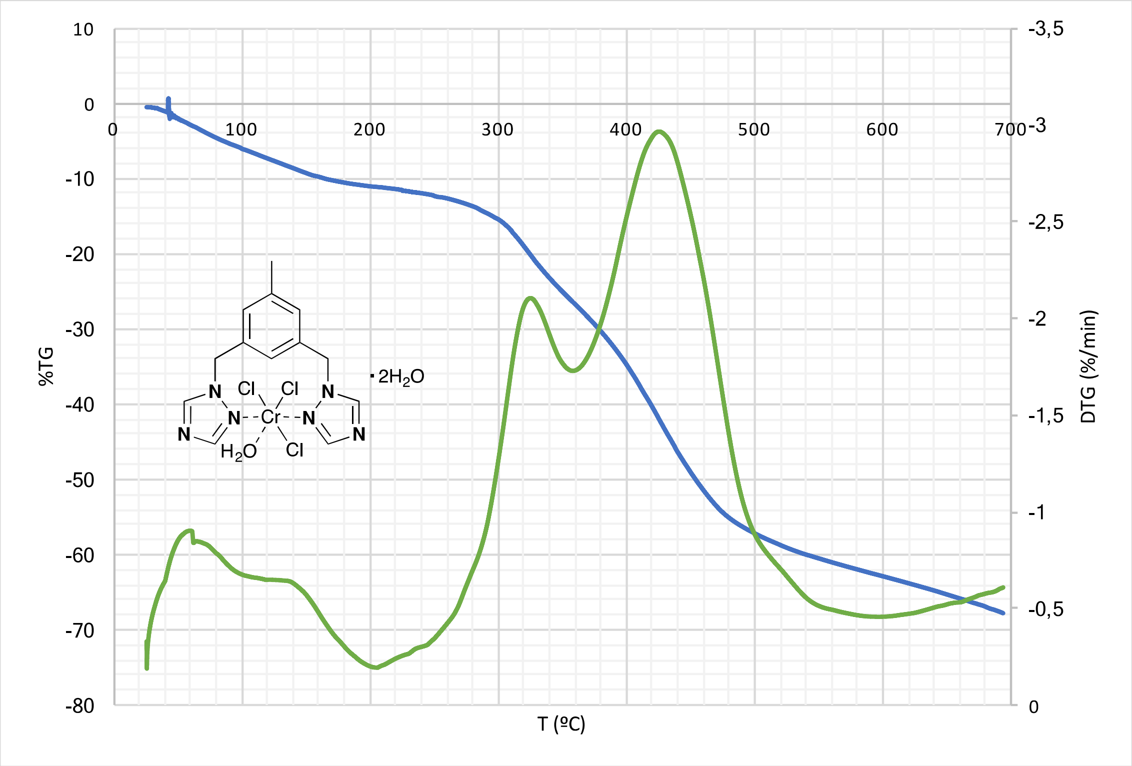

Figure S31. TGA and DTG of (**7**).

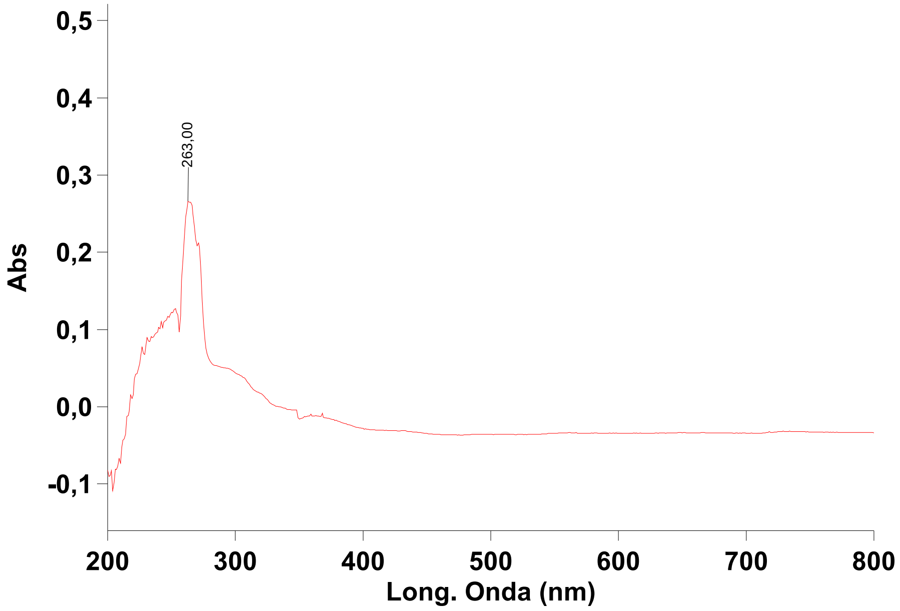

Figure S323. Absorption spectrum of (**L1**) in DMSO (1 mM).

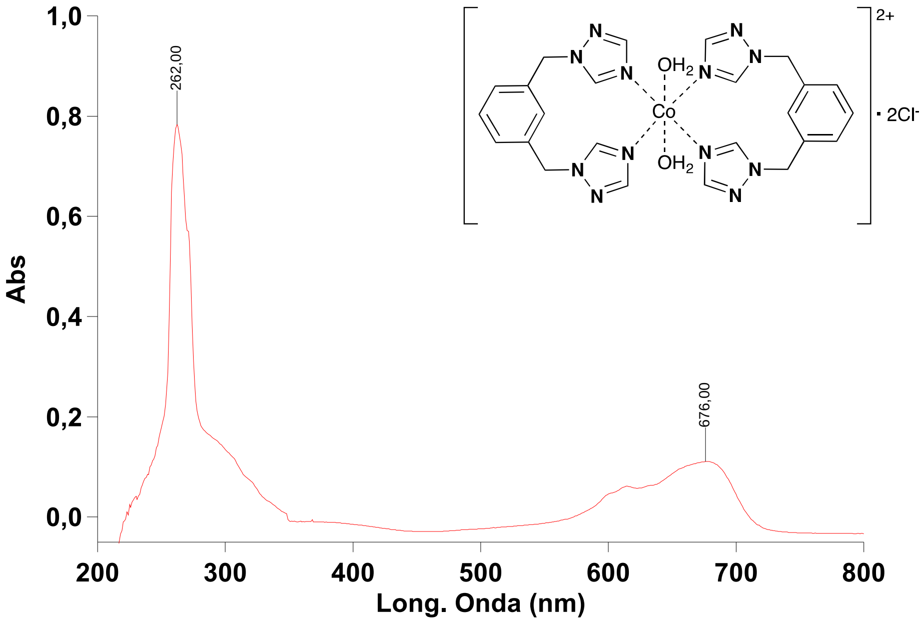

Figure S334. Absorption spectrum of (**2**) in DMSO (1 mM).

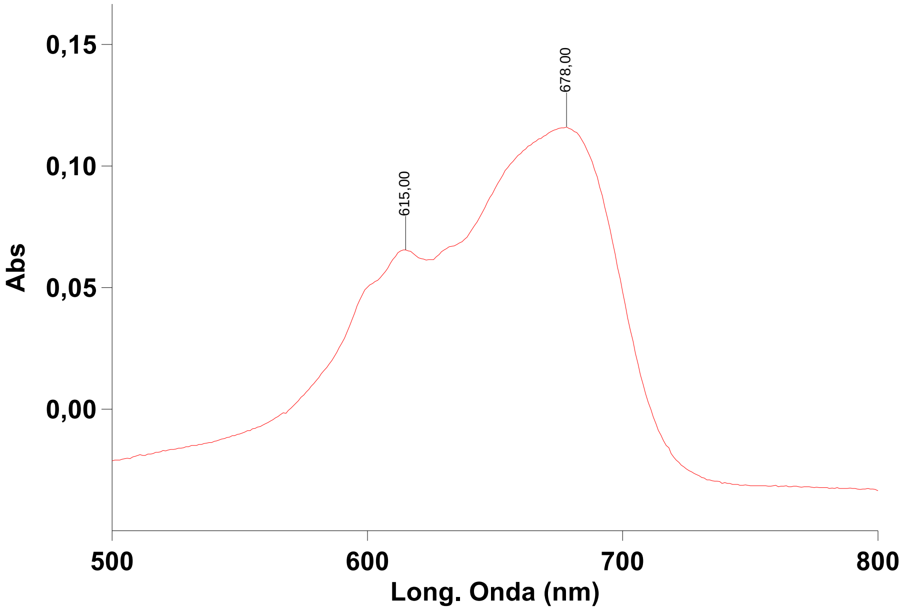

Figure S345. Absorption spectrum of (**2**) in DMSO (1 mM) zone 500-800 nm

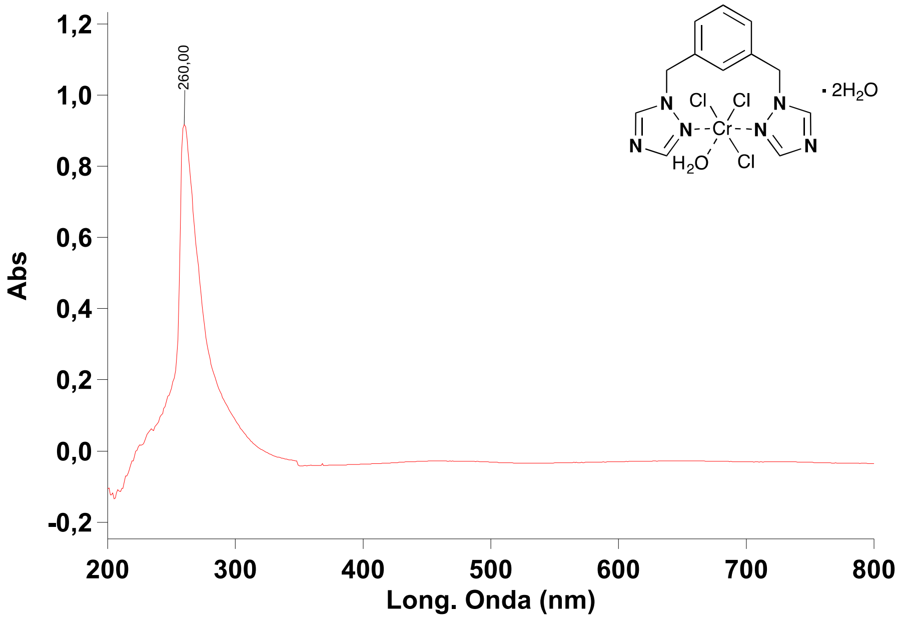

Figure S356. Absorption spectrum of (**5**) in DMSO (0,25 mM).

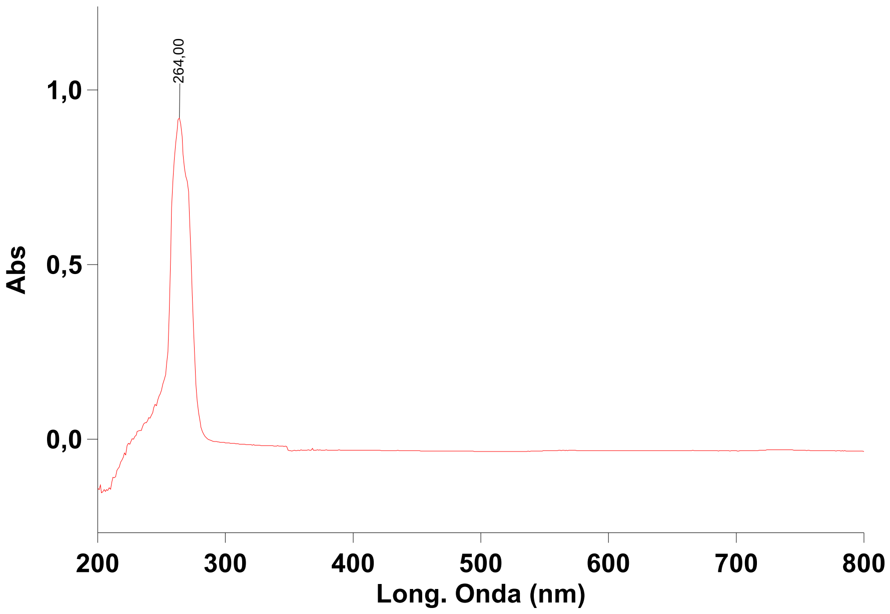

Figure S367. Absorption spectrum of (**L2**) in DMSO (0,21 mM).

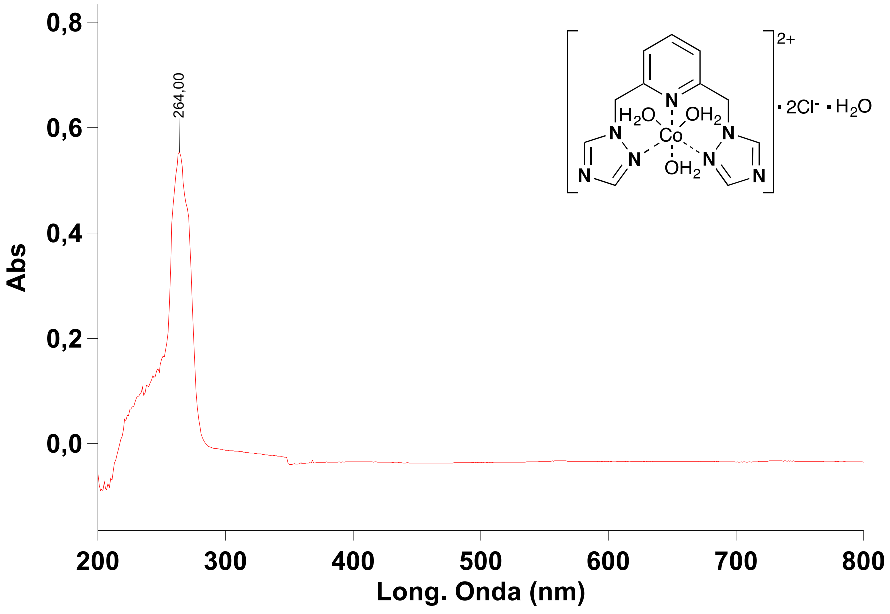

Figure S37. Absorption spectrum of (**3**) in DMSO (0,17 mM).

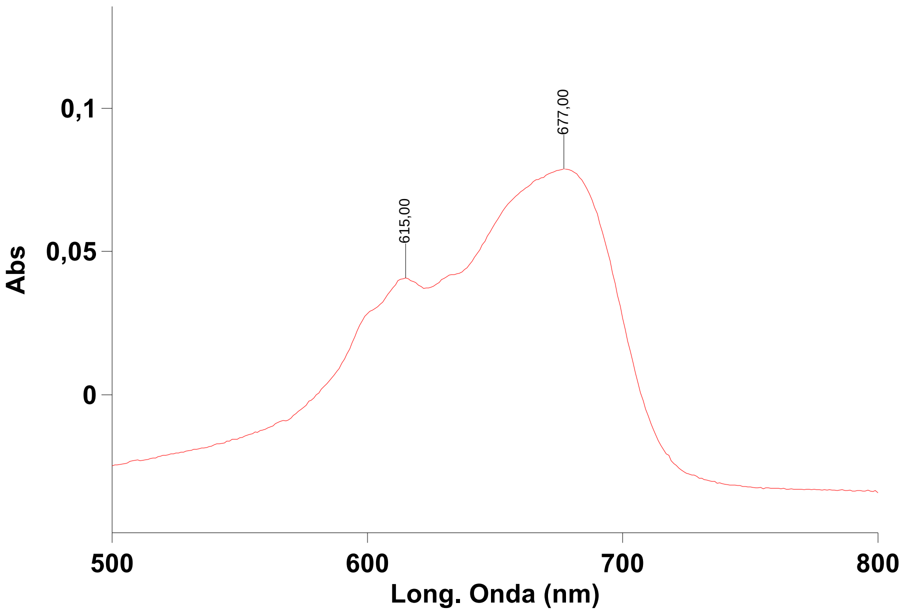

Figure S389. Absorption spectrum of (**3**) in DMSO (1 mM); zone 500-800nm.

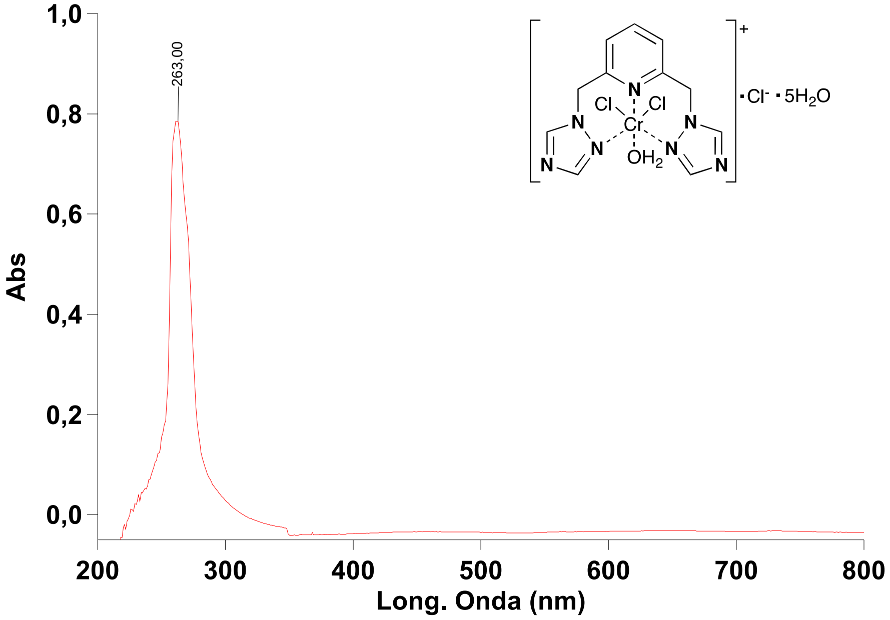

Figure S40. Absorption spectrum of (**6**) in DMSO (0,11 mM).

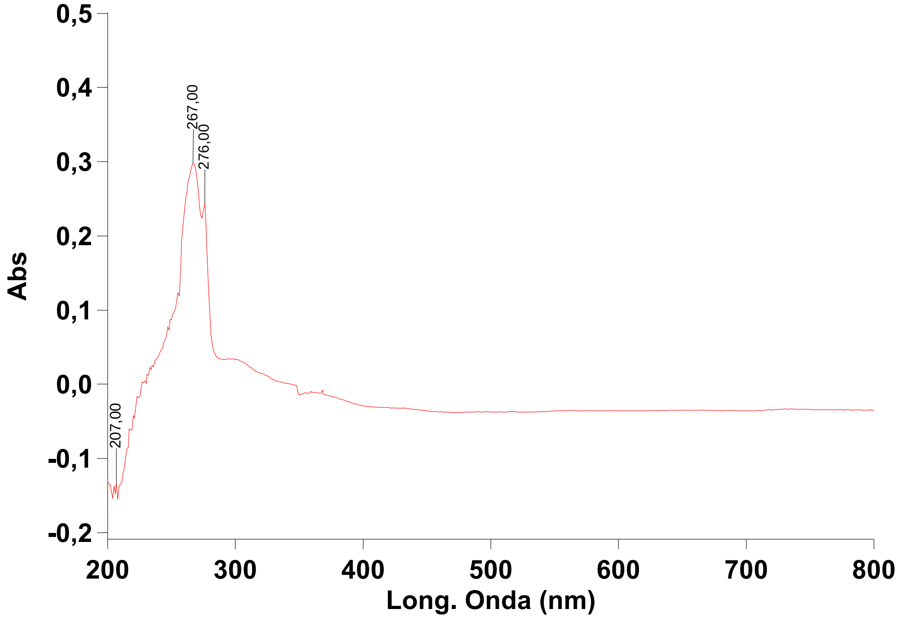

Figure S41. Absorption spectrum of (**1**) in DMSO (1 mM).

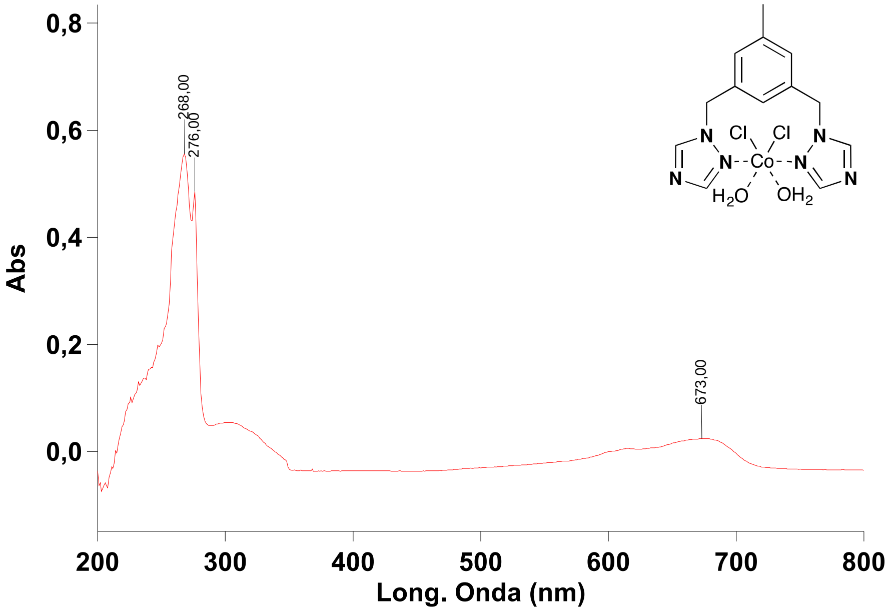

Figure S42. Absorption spectrum of (**4**) in DMSO (1 mM).

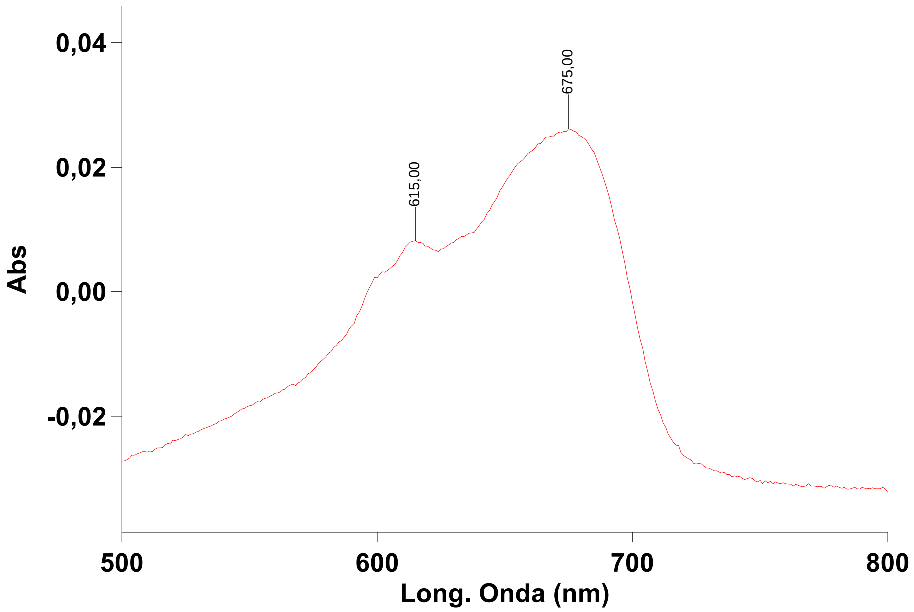

Figure S439. Absorption spectrum of (**4**) in DMSO (1 mM) zone 500-800 nm

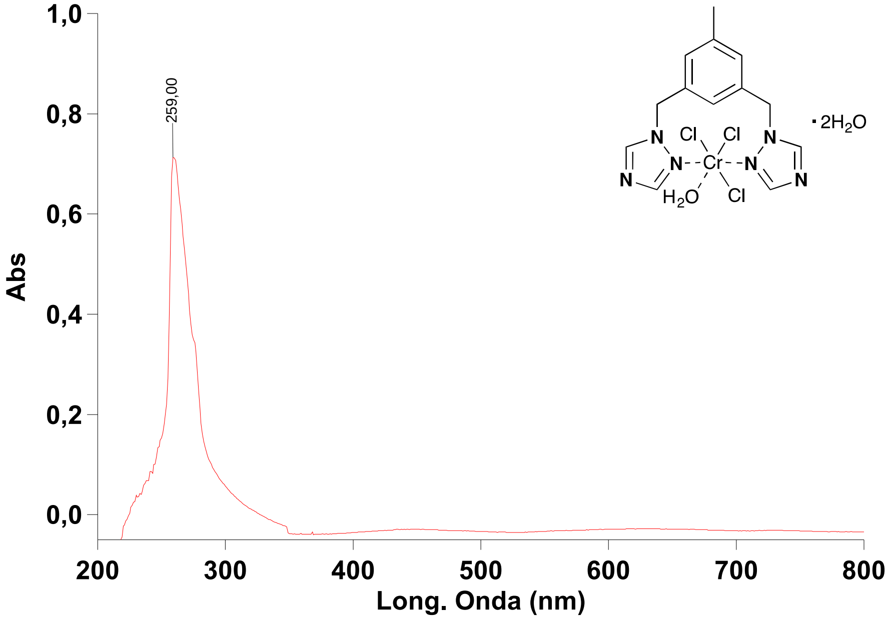

Figure S44. Absorption spectrum of (**7**) in DMSO (0,33 mM).

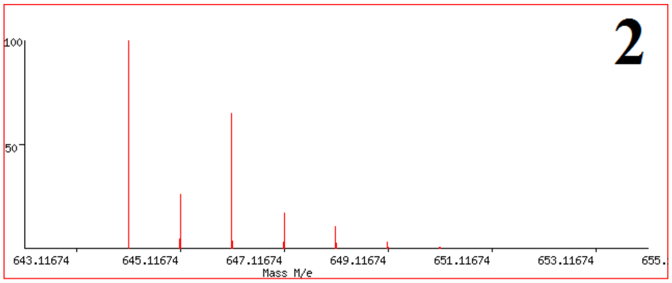
2
100
50
643.11674
645.11674
647.11674
649.11674
651.11674
653.11674
655.
Mass M/e

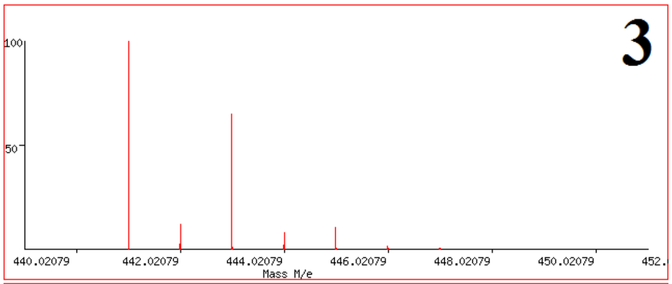
3
100
50
440.02079
442.02079
444.02079
446.02079
448.02079
450.02079
452.
Mass M/e

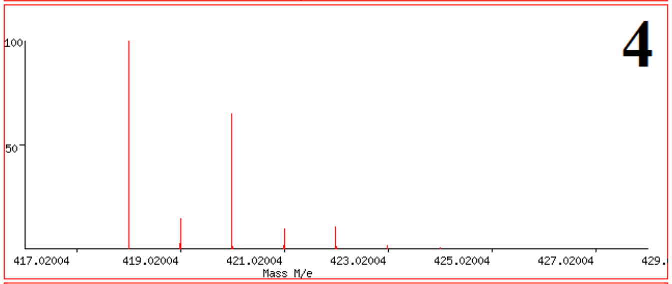
4
100
50
417.02004
419.02004
421.02004
423.02004
425.02004
427.02004
429.
Mass M/e

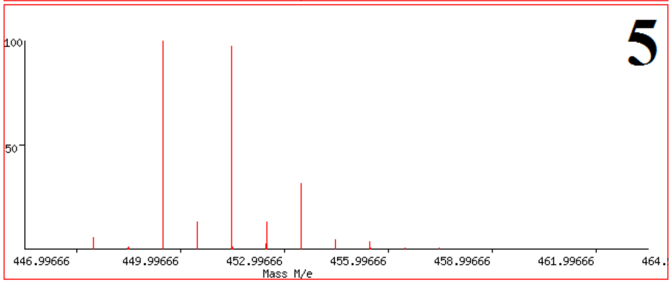
5
100
50
446.99666
449.99666
452.99666
455.99666
458.99666
461.99666
464.
Mass M/e

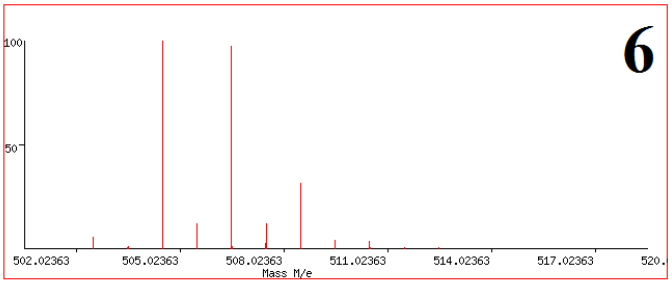
6
100
50
502.02363
505.02363
508.02363
511.02363
514.02363
517.02363
520.
Mass M/e

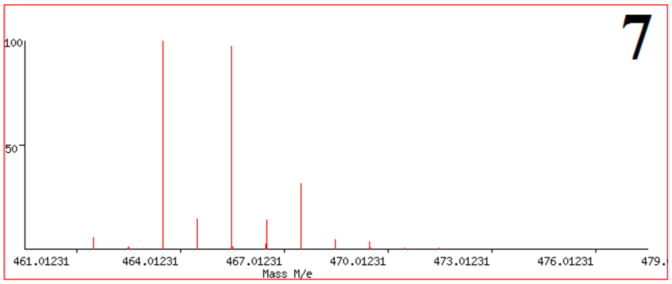
7
100
50
461.01231
464.01231
467.01231
470.01231
473.01231
476.01231
479.
Mass M/e

Figure S45. Mass-spectrum simulation for complexes **2-7**.

Table S1. Specific bands in the FTIR spectra observed for ligands and complexes. Py = pyridine, Tol = toluene, Tz = triazole; vs = very strong; s = strong; m = medium; w = weak; vw = very weak.

Compound	Wavenumber ν (cm^{-1})							
	(O-H)	(C-H)	(C-N)$_{Tz}$	(N-N)	(C-N)$_{Tz}$	tension ring$_{Tz}$		
**L1**	-	3094	1346	1277	1138	891		
**2**	3435	3119	1350	1279	1128	889		
**5**	3429	3124	1348	1283	1123	889		
	(O-H)	(C-H)	stretching ring$_{Py}$	(C=N)$_{Py}$	(C-N)$_{Tz}$	(N-N)	(C-N)$_{Tz}$	tension ring$_{Tz}$
**L2**	-	3105	1574	1512	1462	1273	1141	891
**3**	3402	3119	1576	1524	1460	1277	1125	883
**6**	3379	3130	1578	1533	1460	1283	1125	887
	(O-H)	(C-H)	(C-C)$_{Tol}$	(C-N)$_{Tz}$	(C-N)$_{Tz}$	(N-N)	(C-N)$_{Tz}$	tension ring$_{Tz}$
**1**	-	3090	1609	1466	1346	1261	1134	891
**4**	3412	3111	1609	1464	1352	1279	1128	881
**7**	3375	3130	1612	1462	1348	1285	1125	887

Table S2. Characteristic bands in the Raman spectra of 1,2,4-triazole, **L1**, **2** and **5**.

	Compound			
	1,2,4-triazole	**L1**	**2**	**5**
Wavenumber ν (cm^{-1})	-	1612	1607	1609
	-	1507	1520	1528
	1481	-	-	-
	-	1431	1435	1430
	1376	1367	-	1362
	1360	1341	1349	-
	-	1271	1273	1278
	1258	1237	1244	1237
	1179	1171	1171	1172
	1145	1136	1128	1123
	-	1015	1008	1000
	977	-	-	-
	918	-	-	-

Table S3. Characteristic bands in the Raman spectra of 1,2,4-triazole, **L2**, **3** and **6**.

	Compound			
	1,2,4-triazole	**L2**	**3**	**6**
Wavenumber ν (cm^{-1})	1481	-	-	-
	-	1423	1430	1425
	1376	1364	-	1366
	1360	1346	1348	-
	1145	1139	1125	1121
	-	1091	-	1084
	-	1015	1014	-
	-	991	994	998
	977	-	-	-
	918	-	-	-
	-	588	593	587
	-	525	522	518
	-	346	342	338
	-	-	274	266

Table S4. Characteristic bands in the Raman spectra of 1,2,4-triazole, **1**, **4** and **7**.

	Compound			
	1,2,4-triazole	**1**	**4**	**7**
Wavenumber ν (cm^{-1})	-	1604	1605	1604
	-	-	1520	1530
	-	1496	1464	1472
	1481	-	-	-
	1376	1370	1358	1355
	1299	1286	1282	1283
	1145	1128	1127	1121
	-	1013	996	997
	977	-	-	-
	918	-	-	-
	-	760	759	757
	-	533	522	518

Table S5. Thermogravimetric results (TGA and DTG) for the complexes. n = number of decomposition steps.

Compound (Formula)	TG range / ºC	DTG max/ ºC	n	Mass loss Estimated (calcd.) / %	Total mass loss	Assignment (probable)	Metallic residue
**2**	238-342	301	1	44.60(42.74)	83.85(85.35)	Loss of $C_{12}H_{12}N_6$+ $2H_2O$	CoCl
($C_{24}H_{28}Cl_2CoN_{12}O_2$)	441-585	518	1	22.36(24.44)		Loss of $C_9H_8N_3$	
	586-695	-	1	16.89(18.17)		Loss of $C_3H_3N_3$ + HCl	
**3**	26-156	58	1	3.69(4.06)	64.56(64.81)	Loss of H_2O	$CoCNCl_2$
($C_{11}H_{19}Cl_2CoN_7O_4$)	157-230	184	1	0.97(0.90)		Loss of $2H_2$	
	231-390	315, 347	2	26.27(27,30)		Loss of $C_5H_5N_4$	
	391-493	428	1	11.30(12.18)		Loss of $3H_2O$	
	494-695	-	1	22.33(20.37)		Loss of $C_5H_2N_2$	
**4**	26-298	270	1	18.19(19.99)	62.15(62.82)	Loss of C_2N_3 + H_2O	$CoCNCl_2$
($C_{13}H_{18}Cl_2CoN_6O_2$)	299-338	322	1	7.46(7.85)		Loss of CH_3 + H_2O	
	339-443	352	1	14.21(12.85)		Loss of $C_2H_2N_2$	
	444-619	500	1	17.14(17.85)			
	620-695	-	1	5.15(4.28)			

						Loss of C_6H_3<br>Loss of $CH_4 + H_2$	
**5**<br>($C_{12}H_{18}Cl_3CrN_6O_3$)	26-221<br>222-592<br>593-695	68, 144<br>330, 421<br>-	2<br>2<br>1	11.43(11.93)<br>45.93(46.83)<br>6.20(6.18)	63.56(64.94)	Loss of $3H_2O$<br>Loss of $C_{11}H_{10}N_5$<br>Loss of $C_1H_2N_1$	$CrCl_3$
**6**<br>($C_{11}H_{23}Cl_3CrN_7O_6$)	26-235<br>236-695	63, 139<br>446	2<br>1	13.29(14.18)<br>70.36(68.58)	83.65(82.76)	Loss of $4H_2O$<br>Loss of $C_{11}H_{11}N_7 + Cl_2 + 2H_2O$	CrCl
**7**<br>($C_{13}H_{20}Cl_3CrN_6O_3$)	26-206<br>207-598<br>599-695	66, 122<br>326, 426<br>-	2<br>2<br>1	10.60(11.57)<br>51.49(51.27)<br>5.05(3.21)	67.14()	Loss of $3H_2O$<br>Loss of $C_{12}H_{11}N_6$<br>Loss of CH_3	$CrCl_3$

Ethical issues

All microorganisms used for the biological activity tests were ATCC reference strains classified in the risk group II according to article 67 of resolution 8430 of 1993 of the Ministry of Health of the Republic of Colombia, the cells represented moderate risk for human health and limited for the community. All experiment were performed in accordance with biosafety level II and guidelines established in the decree 351 of 2014 related to the integral management in health care and other activities, as well as the guidelines set out in the Colombian Technical Guide GTC 86, for the implementation of integral waste management.
